# The youth mental health crisis: analysis and solutions

**DOI:** 10.3389/fpsyt.2024.1517533

**Published:** 2025-01-21

**Authors:** Patrick McGorry, Hasini Gunasiri, Cristina Mei, Simon Rice, Caroline X. Gao

**Affiliations:** ^1^ Centre for Youth Mental Health, Faculty of Medicine, Dentistry and Health Sciences, University of Melbourne, Parkville, VIC, Australia; ^2^ Orygen Youth Health, Parkville, VIC, Australia

**Keywords:** youth mental health, anxiety, depression, time trends, prevalence, risk factors

## Abstract

**Background:**

Since the mid-20th century, mental illness has become a leading cause of health burden, particularly among adolescents and emerging adults, with most disorders emerging before the age of 25. Over the past two decades, mental ill health has surged to alarming levels, with evidence confirming that the increase is not just due to better awareness or diagnosis but reflects a genuine public health crisis.

**Study design/method:**

We explore the evolving landscape of youth mental health and its contributing factors, including family dynamics, educational pressures, climate change, social media, and socio-economic challenges, potentially linked to neoliberal policies. A narrative review methodology was employed to analyze these factors and their role in the trends of mental ill-health among young people.

**Study results:**

We document mental health trends since the mid-1990s, focusing on mental and substance use disorders among young people and their current needs. Potential new explanatory factors and megatrends, potentially flowing from a paradigm shift in the global political economy which has largely passed under the radar, yet which has produced fragmentation and inequality, are identified, with the COVID-19 pandemic further intensifying these trends. We discuss methodologies to estimate the contribution of these megatrends and outline potential barriers to implementation, along with strategies to overcome them.

**Conclusion:**

This review calls for a comprehensive global action plan, emphasizing prevention, early intervention, and improved treatment strategies. In addition to strengthening prevention, which may take time and be elusive, immediate action is needed to innovate and expand services, which are currently under-resourced and overwhelmed.

## 1 Introduction

### 1.1 The youth mental health landscape and time trends over the past 30 years

This review is anchored in Rutter and Smith (1)’s seminal monograph, which highlighted an increasing prevalence of youth psychosocial disorders since the mid-20th century. The authors examined the extended transition period from puberty to mature adulthood in the mid-twenties and explored various candidate variables to explain the rising prevalence. Despite their extensive analysis, they found it challenging to draw definitive conclusions. Ultimately, they attributed the trend more to increasing family discord and heightened expectations, particularly related to employment than to other potential factors such as mass media, migration, social disadvantages, unemployment, poor physical health, and declining moral values.

**Figure 1:**
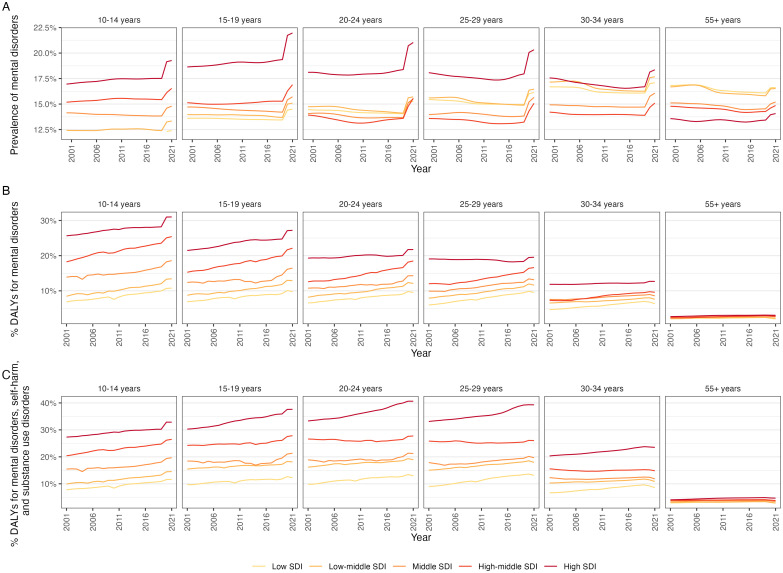
Trends of the burden of mental disorders by age group, country Socio-Demographic Index (SDI) estimated from Global Burden of Disease Study 2021 (2) **(A)** Prevalence of mental disorders; and **(B)** Percentage Disability-adjusted life year (DALYs) attributable to mental disorders, **(C)** Percentage DALYs attributable to mental disorders, self-harm and substance use disorders.

However, the trend of mental health prevenance and burden depends on many factors, e.g., geographical region, socio-demographic status of the region, gender and age group as well as how the prevenance and burden are measured. For example, according to the 2021 Global Burden of Disease Study (2), the prevalence of mental disorders remained relatively stable or decreasing in most countries except for young people 10-19 years in high and high-middle Socio-Demographic Index (SDI) countries prior to the COVID-19 pandemic, which resulted in increases in all age groups across all SDI categories, see **Figure 1A**. However, the percentage of the total burden attributable to mental disorders, see **Figure 1B** or mental disorders, self-harm and substance use combined, see **Figure 1C** increases gradually among those aged 10-34 across most of the SDI categories highlighting the substantial contribution of mental health disorders and self-harm to overall health loss in these populations.

While depression and anxiety can impact people of all ages (3–5), youth aged 10–24 are at a higher risk of experiencing these and other mental disorders (6–9). The pathway to emerging adulthood, from early adolescence to young adulthood, is a time of significant biological, social and personal change that has evolved over generations (10). This period is marked by major life milestones and transitions that coincide with the peak onset of mental disorders (11). Lost productivity at this life stage due to mental illness poses a significant psychosocial and economic burden (12). The imperative to address the mental health needs of young people has been recognised and significant reforms have commenced in some high-income countries to improve mental health service provision for this population (13, 14). These efforts are modest and insufficient in proportion to the scale of need and rapidly rising rates of youth mental ill-health that have been exacerbated by the COVID-19 pandemic (15).Until recently, the evidence for a further increase in youth mental ill-health since the mid-1990s was mixed (16, 17); however, there are increasing indications that this rise is real and alarming in scale (18, 19). Australia’s national survey of mental health recently found that the 12-month prevalence of mental disorders (i.e., anxiety, affective and substance use disorders) among young people had increased by 50% over the past 15 years, disproportionately affecting females (20). The prevalence of depression among young adults in Australia has more than doubled over the past 14 years, according to data from the 2007 and 2020–21 National Survey of Mental Health and Wellbeing (21). The 12-month prevalence of affective disorders, including major depressive disorder, dysthymia, and bipolar disorder, increased significantly from 6.3% to 13.6% among those aged 16–24, and from 7.9% to 11.0% among those aged 25–34, between 2007 and 2020–21 (21). However, according to Liu et al. (22)’ s secondary data analysis of the 2019 Global Burden of Disease study, the annual incidence of anxiety disorders in children and adolescents (10-19 years) decreased by 2.2% from 1990 to 2019, a trend observed primarily before COVID-19 and largely driven by reductions in middle- to low-income countries. There were significant variations in the age-standardised incidence rate (ASIR) and trends among countries. Despite a global decrease in adolescent anxiety disorders over the past 30 years, developed countries continue to see a steady increase in both incidence and disease burden. For example, Portugal had the highest ASIR, while Mexico saw the largest increase. In 2019, Portugal reported the highest number of DALYs (1001.71 million) followed by Ireland (897.80) and Iran (873.64), and India the lowest (212.09 million) (22). Regarding eating disorders among young people, referrals to child and adolescent services in the UK have nearly doubled (23).

Mechanisms underlying this surge remain poorly understood; however, it is likely influenced by a broad range of social and economic changes or “megatrends” (e.g., increasing financial insecurity, family life, educational experiences, and rising health and social inequality) (24). In this review, we focus on the transitional life stage of youth (adolescence and young adulthood). We examine mental health trends relevant to anxiety, depression, psychological distress and wellbeing since the mid-1990s and explore potential explanatory factors associated with prevalence changes.

## 2 Methods

A literature search using Medline (1946), PsycINFO (806) and Embase (1947) was conducted from inception to May 2023. The search strategy included subject headings and free text terms: (anxiety OR depression OR mental disorders OR mental health OR mental illness OR psychiatric disorders OR psychopathology) AND (youth OR young people OR young person OR young adult OR adolescence OR teenage) AND (epidemiology OR prevalence OR time trends). A new search was carried in June 2024 to include new publications: Medline (262), PsycINFO (175) and Embase (281). This search strategy included the aforementioned terms as well as additional terms such as psychosis, eating disorder, and bipolar disorder. Both the search strategies were combined with additional search terms for various sections of this review: causal explanation, determinants, economy, inequality, politics, political leadership, climate change, terrorism, urbanization, globalization, education, employment, unemployment, labour market, structural change, social change, social media, internet, technology, society, trauma, victimization, development, individualism, materialism, expectations, family, culture, COVID, COVID-19, 2019-nCoV, SARS-CoV-2, coronavirus, and corona virus. Searches were augmented by Google Scholar, reviewing the reference lists of relevant studies, and by seminal publications known to the authors.

Primary or secondary studies were selected if they included young people aged 12–25 years and examined trends relevant to anxiety and depression, psychosis, suicidal behaviours/thoughts, eating disorders (ED), Schizophrenia and mood disorders, with at least one time point after 1995 (i.e., following the publication of Rutter and Smith’s monograph). These included studies reporting trends in relation to psychological distress, psychological wellbeing, and somatic, internalising and emotional symptoms. Studies using administrative or health service data were excluded. Two reviewers selected articles identified from the database searches, with one reviewer covering the period from inception to May 2023 and the other from May 2023 to June 2024. Results were summarised according to trends in youth mental health and potential explanatory factors associated with prevalence changes. The percentage of increase or decrease in prevalence is reported when these data were available.

The searches up to May 2023 returned a combined total of 12,047 results after duplicates were removed. Of the 177 full-text papers retrieved, 72 met the criteria for inclusion and were included. The new search strategy yielded a combined total of 373 after duplicates were removed. Out of 122 full-text papers retrieved, 11 met the inclusion criteria, bringing the total number of included studies to 83. Six additional studies were identified following the database searches, thus a total of 89 studies. The characteristics and findings of the studies are shown in **Supplementary Table S1** (See **Supplementary File**). A time-trends graph of overall youth mental health disorders was created using the most recent year of observation in the studies reviewed in this paper. Youth mental health trends were categorized as increasing, stable/mixed (e.g., diverging trends for different disorders/symptoms), or decreasing. Due to small sample sizes, it was not possible to further subdivide the data by disorder or by year.

## 3 Results

### 3.1 Youth mental health trends

Of the 89 studies included, 33% (n=29) were based in Europe, 29% (n=26) in North America, 13% (n=12) in the UK, 7% (n=6) in Australia, 6% (n=5) in Asia, 2% (n=2) in New Zealand, 1% (n=1) in each of South America, and Iran, and 7% (n=6) were multinational. Only 4% (n=4) were undertaken with populations from low- and middle-income countries. 79 (89%) were primary studies, of which 60 (76%) were nationally or geographically representative.

Most studies (80%, n=71) reported an increase in at least one outcome related to anxiety, depression, psychological distress, wellbeing or emotional, internalising or somatic symptoms, psychosis, suicidal behaviours/thoughts, eating disorders (ED), Schizophrenia and mood disorders (**Supplementary Table S1**). The remaining studies reported a decreasing trend (7%, n=6) or no increase (13%, n=12). Trends according to type of mental health problem suggest rising rates of anxiety (15/18 studies), depression (26/37 studies), emotional or internalising problems (20/27 studies), somatic symptoms (8/9 studies), psychological distress (9/14 studies), low mental wellbeing (7/9 studies), suicidal behaviours/thoughts (3/3 studies), Psychosis (1/2 studies), ED (1/1 studies), and Schizophrenia and mood disorders (1/1 studies) (**Supplementary Table S1**). Methodological differences between studies (e.g., outcome measures, sampling method and sample characteristics) are a probable explanation for the variation in trends.

The degree of increase varied within and across nations. The proportion of young people experiencing anxiety increased by 29–84% in the US (25, 26), 77–164% in Sweden (27, 28), 105% in Australia (20, 29), and 115% in Canada (30). Severe anxiety increased by 86% in the US (31) and high levels of anxiety doubled in Iceland (32). For depressive symptoms or disorder, the percentage of increase was 8%–119% in the US (33–42) and 87% in Australia (43). Severe depression rose by 145% in the US (31), while the change was modest in Finland (+18%) (44). The proportion of young people with emotional or internalising problems increased by 25–79% in the Netherlands and Nordic countries (45–48), 43% in Canada (49), 48% in New Zealand (50), 55–61% in the UK (51, 52), and 10%-64% in Poland (53, 54). For psychological distress, the percentage of increase was modest to large: 43–135% in the UK (55–58), 12–70% in the US (33, 37), and 7%-47% in Ethiopia (59). In the UK, severe psychological distress tripled (58). Somatic symptoms increased by 25–54% in the Netherlands (45, 48), while low wellbeing rose by 119% in the US (31) and 118% in Denmark (60). Rates of suicide increased from 3.6- 7.1 (per 100, 000) in Asian American and Pacific Islander (AAPI) youth in the US (61), ED increased by 5% per month from 2017-2021 (62), and Schizophrenia and mood disorders increased by 4% per month (62).

There are indications that trends have risen since 2011–2016 for psychological distress, low mental wellbeing, and depressive and anxiety symptoms (6, 31, 32, 34, 39, 41, 42, 58, 63–67). Since 2020/the COVID-19 pandemic, rates of psychological distress, anxiety and depressive symptoms have increased further, and mental wellbeing has worsened (56–58, 60, 68–70).

Increasing trends were not observed in Canada, New Zealand, England, China, Netherlands, and Chile for depression; Belgium and Japan for psychological distress; Poland for somatic symptoms; the UK for low wellbeing; and Scotland, Switzerland and Iran for internalising symptoms. However, for each of these locations (except the UK and Canada), only one study was available for the corresponding outcome. Where declining trends were reported, the percentage of decrease was 22% for depression (71) and 6–29% for distress (72, 73).

Trends in relation to gender suggest greater increases among females for anxiety, depression, distress, emotional problems, psychosis, and internalising symptoms (31, 34, 37, 39, 63, 74–78). However, some studies have reported increases for both genders in relation to anxiety, depression and emotional problems (28, 38, 49, 51, 54, 77, 79, 80) or higher increase for males for psychological distress (81), and low mental wellbeing (82). These discrepancies across similar outcomes likely reflect methodological heterogeneity, particularly in terms of outcome measure, which varied across studies. Also, outcomes of this review may not have detected non-specific symptoms (e.g., irritability, anger, and risk-taking) that may represent gender-specific markers of distress in young men (83).

**Figure 2:**
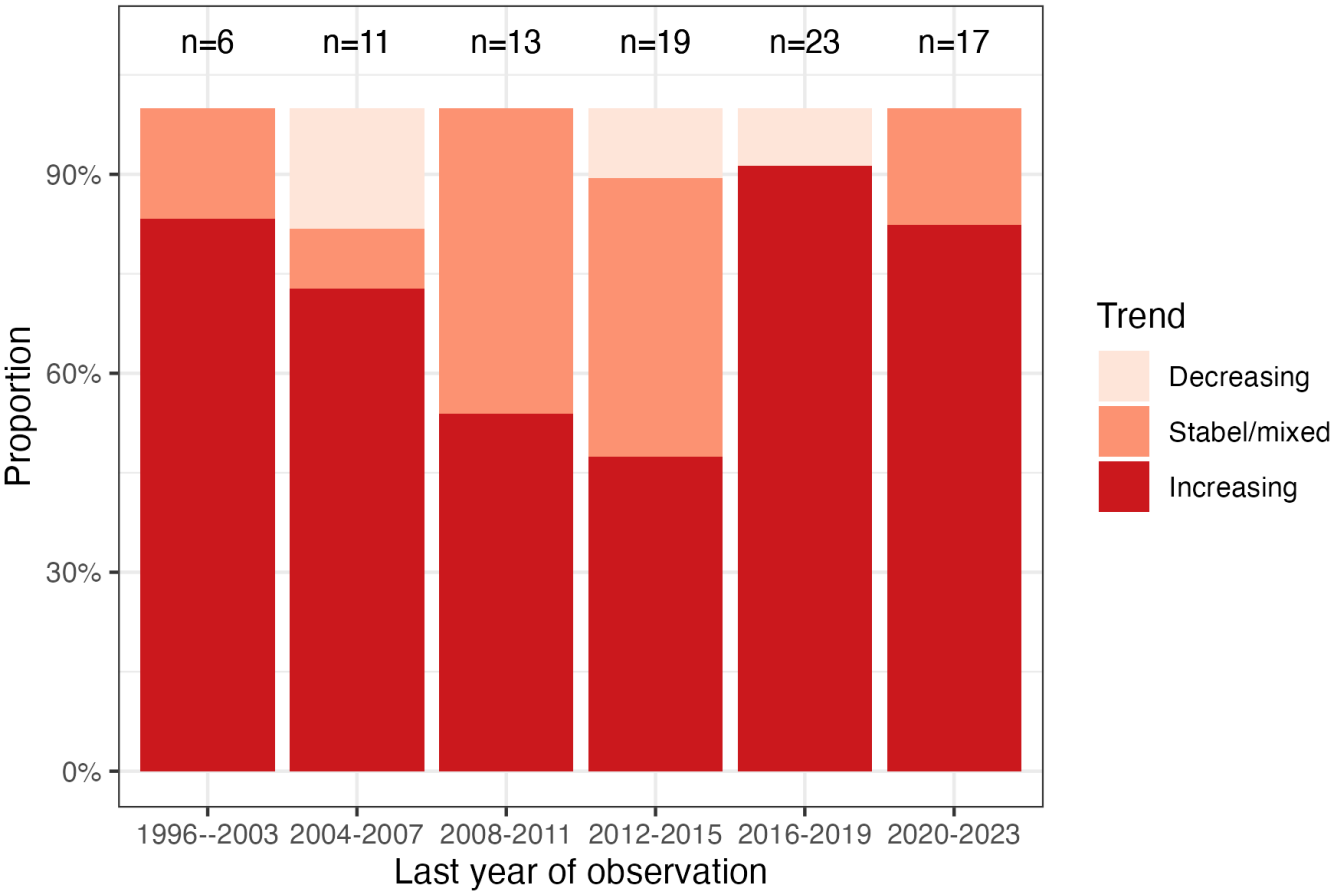
Time trends of youth mental health disorders from 1996-2023.

**Figure 2** illustrates the trends in youth mental health disorders from 1996 to 2023 (the most recent years of observation in the studies reviewed). The figure shows a declining trend from 1996 to 2015, followed by an increase starting in 2016. The effect of the COVID-19 pandemic (2020-2022) is unclear from this figure at least, and it appears that the increase from 2016 is difficult to explain on that basis.

### 3.2 The social, economic, and commercial determinants of mental health

Social determinants of mental health refer to the structural conditions individuals are exposed to throughout their lives, from conception to death (86). These conditions affect mental health outcomes and contribute to disparities within and between populations. Key factors include income, employment, socioeconomic status, education, food security, housing, social support, discrimination, childhood adversity, neighbourhood conditions, and access to affordable health care (87–89). The likelihood of encountering protective or harmful social determinants is influenced by the distribution of money, power, and resources at global, national, and local levels, shaped by policy choices (86).

The impact of mental health issues is particularly evident among young people. Young people have high rates of self-harm, and suicide is a leading cause of death among them (90, 91). Poor mental health is closely linked to other significant health and developmental concerns, such as educational achievements, substance use and abuse, violence, and reproductive and sexual health (37). Poor mental health can hinder life chances, impacting both lifespan and quality of life. Therefore, improving young people’s mental health by addressing modifiable social risk factors through effective prevention strategies is a critical social justice issue.

Commercial determinants of health, a relatively new area of study, highlight how corporate actions, conditions, and omissions significantly impact health (92). Young people are especially vulnerable to advertisements and celebrity endorsements. For example, marketing electronic nicotine delivery systems and cannabis to young people can activate sensitive and developing pathways in their brains (93). On the positive side, the private sector can enhance public health by providing living wages, paid parental leave, sick leave, and health insurance, all of which contribute to better health outcomes (93). However, as highlighted by critics like Pennington (94) and Monbiot (95), in the context of neoliberal political and economic settings, with minimal regulatory safeguards and weakened central government, the private sector now falls increasingly short in delivering these benefits equitably, leading to rising inequality and growing disparities in health outcomes.

A range of social, environmental, and commercial factors influence the collective life experiences and mental health of young people and their families (93, 96, 97). Ensuring that young people have a healthy environment where they can grow, participate actively in their communities, and pursue goals that honour their uniqueness and dignity is essential (98). However, this is a complex challenge, as meeting basic needs such as safety, education, financial stability, healthy caregivers, and positive life experiences is fundamental to building health and resilience (99, 100).

#### 3.2.1 Dynamic new megatrends

The identification of mechanisms underlying trends in youth mental health is an emerging area of research. Most studies to date have examined correlation rather than causation. An exploratory review of potential proximal and distal factors associated with trends is provided in the following sections. Proximal factors reflect those within the young person’s immediate environment: developmental changes, family, education, health and lifestyle, social media, childhood trauma, and bullying (86, 98). Distal factors reflect broader societal contexts, which are often related to and mediated by proximal factors: economy, socio-economic disadvantage and inequality, politics, and climate change (86, 101). This review considers the influence of both proximal and distal factors, largely related to Western and high-income countries, structuring the discussion based on Kirkbride (86)’s framework of social determinants. This framework includes individual-level social determinants (i.e. proximal) and broader environmental determinants (i.e. distal).

##### 3.2.1.1 Individual level social determinants

###### 3.2.1.1.1 Developmental trends

This section explores developmental trends that influence youth mental health, including factors such as family environment, education, health and lifestyle, experiences of abuse or neglect, bullying and cyberbullying, and the role of social media.

###### 3.2.1.1.2 Family environment

With respect to family structure, while there has been an increase in the proportion of young people not living with both biological parents, this has not been associated with increasing rates of psychological distress (72, 102), depressive symptoms or disorder (43, 79), and emotional problems (76). On the other hand, it may have retarded the maturational process of attaining independent identity and adult status. Parent-adolescent arguments have been found to explain a large proportion of the rise in self-reported adolescent psychological distress, although reverse causality could not be eliminated and the survey items used to measure arguments differed at the two time points (102). Based on two large national surveys using identical measures of adolescent and parent emotional problems over a 20-year period, increases in maternal emotional problems were found to modestly contribute to increasing rates of adolescent emotional problems (103). This was based on three key findings: maternal and adolescent emotional problems increasing at the same time; parental emotional problems prospectively predicting offspring emotional problems, beyond reverse causation and influences of shared adversity; and cohort differences in adolescent emotional problems were attenuated when controlling for trends in parental emotional problems. Armitage et al. (104) posits that these trends in family structure have not significantly impacted trends in youth mental health, as similar increases in adolescent emotional problems are observed across different family structures (103, 105, 106). While they may not account for the rise, family environment and structure are still an important influence, and equally, efforts to alleviate childhood poverty, food insecurity, and family homelessness are critical for addressing these social determinants of mental health among young people.

###### 3.2.1.1.3 Education

Educational reforms in the UK and many other countries have led to increased grading and testing, which significantly contributes to student anxiety and stress (104). Studies report rising school-related stress, academic pressure, and burnout among adolescents, with notable variation across countries (107–111). Female adolescents are particularly affected, showing increased worry about academic performance, contributing to a widening gender gap around 50% for internalising problems (102, 112). Studies that have compared trends between students and non-students indicate variation across countries and genders. A meta-analysis across 57 nations found an overall increase in anxiety among both students and non-students between 1970 and 2010; however, when analysed according to country (US, Australia, UK, Canada), only students in the US showed a significant increase (113). Recent data from England (2000–2014) suggest that only female students have shown increases in common mental disorders, although the sample was relatively small (114). There is little experimental evidence as to why rates have increased among some students, particularly females. Hypotheses include rising tuition fees, student debt (113, 114) and increasing educational expectations or pressures that have impacted females (75, 108, 115), which when combined with other stressors (e.g., concerns about personal identity), has led to an accumulation of risk factors for mental ill-health (115). While these trends indicate increased anxiety and mental health issues among students, particularly females, the evidence is complex and varies across countries, necessitating further research to unravel the underlying causes, causal sequence, and implications.

###### 3.2.1.1.4 Health and lifestyle

Indicators of health that are commonly associated with mental ill-health have similarly declined; however, their impact on youth mental health trends is unclear. The vaping epidemic exemplifies how social factors impact substance use, with electronic nicotine delivery systems (ENDS) popular among youth due to targeted marketing and social media influence (116, 117). Youth with mental illness may use ENDS to manage symptoms or medication side effects, contributing to conditions like depression, ADHD, and conduct disorder (93). Sleep disturbances have risen, which has been linked to electronic device usage (118). Physical activity has declined, especially since the COVID-19 pandemic (119). While obesity rates have risen, this has not been associated with increasing rates of distress (102). There has been a notable increase in the percentage of females in the clinical range for mental health and substance use among Finnish young people (120). The effect of urbanisation on anxiety and depression trends is mixed and limited by differences in measures of urbanisation and the conflation other outcomes (e.g., conduct disorders) (49, 53).

In contrast, several studies have found that some lifestyle trends have positively improved for adolescents, with signs of decreasing alcohol consumption, illicit drug use, and risky behaviour (47, 121–125). While alcohol use has declined among Generation Z, there has been a rise in the use of substances such as cannabis, which is now more potent and readily available (126, 127). An increase in depressive symptoms in Norway was partly attributed to increases in cannabis use and disordered eating (79).

###### 3.2.1.1.5 Abuse or neglect experiences

At present there are no direct links between population changes in youth mental health and exposure to violence, abuse and neglect. While childhood maltreatment is common and remains a major public health concern, stable (128) and, in some instances, decreasing trends in exposure to violence and abuse (122, 129–131) cautiously indicate that these factors may not be likely candidates to account for the rise in prevalence of disorders. However, this requires robust investigation across the various types of maltreatment as increases in emotional abuse and neglect have been reported (132).

###### 3.2.1.1.6 Bullying and cyberbullying

Studies indicate that the impact of being bullied surpasses the effects of other childhood adversities and adult abuse (133, 134). While bullying victimisation has shown a downward trend in most European countries (135, 136), increasing trends have been observed in the UK (135, 137). In Scotland, increasing rates of victimisation have been linked to a decline in the mental wellbeing of adolescents (137). While cyberbullying victimization has remained stable (136) or increased among adolescents (138–140), it has negatively impacted mental health. This suggests that both bullying and cyberbullying victimization are indicators of comorbid mental health problems (136). Conversely, a rise in cyberbullying in 2020 occurred in the absence of increasing anxiety and depressive symptoms (141). According to Patchin and Hinduj (142), the proportion of individuals who have experienced cyberbullying at some point in their lives has more than doubled over the years. Protecting youth from bullying and its detrimental effects is crucial, necessitating safe, respectful, and inclusive school environments that do not tolerate such behaviour.

###### 3.2.1.1.7 Digital media/social media

The digital world offers youth avenues for connection, creativity, and support, but also carries significant risks. Smartphone usage may contribute to sleep deprivation (143), and social media addiction may further exacerbate mental health issues among young people (144). While evidence suggests a longitudinal association between social media and mental health in young people, the magnitude is likely to be small and the nature of this association is uncertain owing to bidirectional effects and the range of mediating factors (145–149). With these considerations, recent studies have suggested a link between increased social media/electronic device usage and both a decrease in psychological wellbeing and increase in depressive symptoms, occurring between 2011/2012 and 2015/2016 (63, 64). This was based on two criteria: social media/electronic device usage being correlated with higher depressive symptoms/lower wellbeing and usage significantly increasing simultaneously with depression/low wellbeing. These findings are limited by the broad categories used to measure usage (e.g., ‘almost every day’), which do not capture the variance in this variable given that the majority of young people use social media daily (150). Other studies using the same surveys analysed by Twenge et al. indicate a small correlation between social media usage and both depressive symptoms and wellbeing (150–152) and that social media explained only 4% of the increase in depressive symptoms (150). Furthermore, between 2009 and 2017, the association between social media usage and depressive symptoms was found to be weak and confined to only 2009–2010 (152).

##### 3.2.1.2 Wider social environmental determinants

###### 3.2.1.2.1 Economic trends

This section explores economic trends, including the labour and housing markets, socio-economic disadvantage and inequality, climate change, and political factors, all of which impact youth mental health.

###### 3.2.1.2.2 Labour market/unemployment

The impacts of the 2008 global financial crisis on young people were substantial and included insecure employment and income (153–157). These trends have relevance to the COVID-19 pandemic (see below). In Southern Europe, cross-sectional repeated surveys spanning before and after the 2008 recession showed that symptoms of anxiety and depression increased among students (potentially due to limited employment prospects) (158) and that these symptoms among young people have become more strongly associated with insecure employment (e.g., fixed term contracts) following the recession (159). Across the working-age population, young Australians (15–24 years) have shown the largest decrease in mental health and wellbeing and increase in high/very high symptoms of psychological distress since 2010 (160). This coincided with an increase in insecure employment, inadequacy of pay, and the casualisation of the youth labour force.

In the US, no association was found between rising adolescent depressive symptoms and the overall unemployment rate immediately after the 2008 recession (63). However, when labour market trends have been measured specifically in relation to young people, an association has been found between increases in the proportion of young people not in the workforce and increases in mental health symptoms among adolescents (feeling low, headache and sleep difficulties) between 1983 and 2005 (161). This lends some support to the finding that proximal (i.e., adolescents’ worry about family finances) rather than societal level factors (i.e., overall municipality unemployment rate) are more strongly associated with increasing psychosomatic symptoms among adolescents during a period of economic downturn in Sweden (5). However, the direction of causality remains unclear. As somewhat expected, little evidence was found to support the role of economic variables (e.g., no working parent, worry about unemployment) in explaining increases in psychological distress in Scottish adolescents during a period of reduced economic hardship (102). Moreover, the pandemic seemed to have affected school engagement among young people. For instance, symptoms of anxiety decreased from pre-pandemic levels to during the first UK lockdown but increased upon the return to school for 13-14-year-olds (162). These findings underscore the complex and context-dependent relationship between economic instability, insecurity of work, youth mental health, and factors like school engagement during the pandemic, highlighting how both direct experiences of economic hardship and broader economic conditions can variably impact mental health outcomes.

###### 3.2.1.2.3 Housing market

Housing market and economic trends have contributed to a generation of young people who frequently face residing with parents for longer or living in unaffordable and insecure rentals (154). Economic downturns negatively impact young people’s mental health, with effects comparable across different economic indicators like unemployment rates and housing prices (163). Golberstein et al. (163), found that a decline in unemployment rates (2001-2013) in the US is associated with a reduction in the severity of mental health problems in children and adolescents (4-17 years), similar to the impact on adult health outcomes. Conversely, a five-percentage point increase in unemployment during the Great Recession was linked to a 35% to 50% increase in clinically significant mental health issues among these age groups (163). Home-ownership rates among young adults have decreased (154, 164) amid stagnant wages, unstable employment conditions and dramatically increasing house prices (165). This has negatively impacted the mental health of adult renters (166). Young people who rent fear that housing security and wealth are unattainable and have reported feeling anxious, depressed and stressed due to insecure, expensive and poor-quality housing, and rising rental costs have added to this precarious situation for many (167).

###### 3.2.1.2.4 Socio-economic disadvantage and inequality

Socio-economic and intergenerational inequalities have been increasing since the 1990s in most high-income countries (168, 169). Today’s young people are not only struggling more than older generations, but they are also worse off compared to previous generations at the same age and are expected to face greater challenges as seniors than the current over-65s (170). A global study by (171), using data between 2009-2022, has shown that relative inequality in youth wellbeing has worsened, with emotional distress and life satisfaction becoming increasingly unequal between socioeconomic groups. This suggests that growing income disparities are intensifying relative inequality across generations, leaving today’s youth more disadvantaged compared to previous generations (171). In the UK, a comparison of three nationally representative cohorts with comparable data revealed a widening disparity in emotional problems according to level of family income, with a fourfold increase for adolescents in the lowest income group between 1974 and 1999/2004 (172). This was partially accounted for by the increasing impact of socio-economic adversity (e.g., insecure housing tenure), mediated by adolescents’ proximal family environment. Increasing social inequalities have similarly been observed in relation to rising rates of depression in Finland and the US (6, 44). In Japan, a nation with higher levels of equality than most others, psychological distress among adolescents decreased significantly from 10.7% to 7.6% between 2007 and 2013 (72). However, the patterns of psychological distress relative to socioeconomic status remained consistent throughout the study period. Adolescents in both lower-income and higher-income households reported more psychological distress compared to those in middle-income households. Conversely, findings from Denmark indicate that the largest increases in emotional symptoms have occurred in high and middle socio-economic classes (46); however, a substantial proportion of cases with emotional problems had missing socio-economic status data, which may have skewed findings. Data from the US suggest increasing rates of major depressive episode in adolescents between 2005 and 2017 across all income levels, although inequalities remained as those in the lowest income group experienced higher annual rates of major depressive episode than the highest income group (37).

The COVID-19 pandemic and its associated economic crisis have exacerbated inequality among vulnerable and disadvantaged groups, including young people (173). During the pandemic, young people have experienced increased rates of educational disruption, unemployment or precarious employment, financial insecurity, and housing stress (173, 174). Young people of lower socio-economic status have experienced greater economic hardship (174). This amplification of socio-economic determinants of mental health and of inequality has likely contributed to an increase in anxiety and depression that has disproportionately affected young people (15, 68, 174–176).

###### 3.2.1.2.5 Climate change

There is increasing evidence of climate change impacts on young people’s mental health who are a priority population group in the context of climate change. These include anxiety, sadness, grief, hopelessness, powerlessness, depression, and existential worries about the future (177–180). A range of factors including government inaction on climate change, social media exposure, and social challenges such as housing instability, food insecurity, and increased conflict influence this relationship between climate change and young people’s mental health (177, 178, 181–183).

According to Sciberras & Fernando (184)’s eight-year longitudinal study which focused on climate change worry among Australian adolescents found that adolescents with high levels of worry related to climate change that are persistent over time showed higher rates of depressive symptoms in late adolescence than adolescents with moderate levels of climate change related worry. Although 50% of young people have experienced a negative emotional response to climate change (185), a causal relationship and time trends between climate-related negative emotions and mental ill-health in young people has yet to be fully established (186). There has been one study that looked at the causal relationship between climate change and mental health. Patrick et al. (183)’s systems dynamics study developed a systems map highlighting the relationships between causal factors (e.g. under theme areas of government, services and structures, personal experience of environmental disasters, and social norms, communication and taking action). While the nature of causal relationships requires further investigation, higher temperatures have been associated with increased suicide rates (187) and lower mental wellbeing (188).

###### 3.2.1.2.6 Politics

Although anxiety surrounding political elections and decision-making is not a recent phenomenon, there is concern about the impact of recent and current political landscapes (189). Both immediate and potential future negative effects on youth mental health have been observed. This includes increased stress and anxiety during the 2016 presidential election in America (190, 191); increasing concern in the UK because of the Brexit vote and its uncertain consequences, particularly on employment and education (192); and social unrest in Hong Kong that has been independently associated with depressive symptoms (193). Gimbrone et al. (194)’s study (2005-2018) highlights a growing mental health disparity (e.g. depressive symptoms) among adolescents based on their political beliefs. The findings suggest that the ideological perspectives through which adolescents interpret the political climate can have varying impacts on their mental well-being.

## 4 Discussion

The findings of this review indicate a substantial rise in youth mental health issues including anxiety, psychological distress, self-harm, suicide, and depressive symptoms, contributing to a global youth mental health crisis as highlighted by a recent Lancet Commission on youth mental health (14). The magnitude of change varied within and across nations and was not observed in some studies. However, in others, it was substantial, with the percentage of increase as high as 164% for anxiety, 135% for psychological distress (increasing to 242% for severe distress), and 119% for both depression and low wellbeing (increasing to 145% for severe depression). An important consideration is whether increasing trends reflect a real population change or are an artefact arising from reduced stigma, improved mental health awareness, greater willingness to disclose mental health issues, a widening of diagnostic thresholds, and population growth (195–197). These factors might contribute to an inflation of prevalence rates by capturing individuals with transient distress that does not necessarily require clinical intervention (198). Additionally, the role of cultural and societal shifts, such as the influence of digital media and the pressures of modern life, may contribute to these trends, making it essential to consider both the real and perceived changes in mental health across generations (199). However, these factors contributing to increasing trends in youth mental health remain unclear and individually only explain a small proportion of the increasing trend and the interpretation of findings and causal inferences are limited by methodological constraints (e.g., unmeasured cofounders or co-occurring social changes, lack of harmonised measures across studies and, in some instances, timepoints) as identified in the review. Although potential shifts in respondent reporting and diagnostic approaches have been difficult to unequivocally refute, Twenge et al. (200) found that increases in psychopathology were not explained by a greater willingness to report mental health problems after controlling for socially acceptable and defensive responding. Further validation of a true rise can also be provided from related rising trends, including increased rates of suicide (28, 63), co-occurring functional impairment (36, 74), and mental health service use (28, 30, 71, 201, 202).

According to the findings of this review, over time, several social and commercial determinants have shown varying trends in their impact on youth mental health. increasing trends are observed in areas such as the instability and identity explorations associated with adolescent and emerging adulthood, homelessness and housing insecurity, cyberbullying, social media usage, and economic precarity, particularly in insecure employment. Conversely, there are decreasing trends in certain substance use, bullying victimization, and exposure to violence and abuse, though the latter also shows some stability. Mixed or stable trends are noted in areas like family structure, educational stress, urbanization, and the impact of maternal emotional problems.

Given the current context of the aftermath of the COVID-19 pandemic, many of these factors, particularly socio-economic inequality and climate change, will likely be exacerbated in the coming years (203, 204). Mental health and social services already need to be urgently expanded to meet the rising needs of young people and offset the likelihood of persistent mental health problems into later adulthood, including early parenthood (13). Hence, there are critical intergenerational consequences of inaction given parental mental health problems are predictive of offspring mental ill-health (205–208).

### 4.1 Determining the relative contributions (underlying forces) of these candidate causes

Adolescence and emerging adulthood have undergone two key maturational shifts: an early onset of puberty and a delayed commencement of independent adulthood (209). This has extended the period of heightened susceptibility to environmental stressors and mental ill-health. This lengthening of adolescence commenced prior to the recorded rise in youth mental ill-health in the 1990s (1). While its impact on more recent trends is unclear, it has converged with educational, labour market and economic shifts (e.g., prolonged years of education; insecure employment, income and housing; and delayed independence) that have contributed to highly precarious pathways to adulthood (113, 159, 163, 167). Emerging adulthood is a heightened period of instability and identity explorations, which have been correlated with anxiety (210). Increasing and cumulative exposure to psychosocial stressors during this period of precarity may have increased the risk of mental ill-health and contributed to deteriorating trends (13, 98, 211). However, the salience and impact of these influences are likely to vary across different cultural settings, as well as in relation to levels of wealth and development around the wealth (212, 213).

Various opinions and theories have emerged to examine and evaluate the candidate causes of negative trends in youth mental health. For example, Twenge (118, 214) and Haidt (143) argue that the rise of social media and increased screen time are the dominant contributors to the deterioration in mental health among young people, suggesting that these factors lead to heightened anxiety, depression, and social isolation. Twenge’s research points to a correlation between the advent of smartphones and a decline in adolescent mental health (215). Haidt (143)’s *The Anxious Generation* proposes a stronger relationship between the rise in social media use and deteriorating mental health among young people, positing that the increased screen time and social comparison are major contributors to the surge in anxiety and depression. While Haidt (143)’s work has brought significant attention to this issue, it has also been critiqued for potentially overemphasising the role of social media at the expense of other sociocultural and economic factors that may contribute to youth mental health trends. Additionally, the reliance on correlational data in Haidt (143)’s argument, limits the ability to draw definitive conclusions about causality. Thus, support for a causal link between social media usage and increasing depressive symptoms and low wellbeing is limited and requires further investigation using more robust measures of social media use. Despite this, politicians in several jurisdictions have been persuaded to implement age linked restrictions on access to the internet and social media for teenagers (216). Such action can be argued to be misdirected, lacking in nuance and to produce unintended consequences (217–219). This regulatory gap has allowed platforms to prioritize engagement and profit over the well-being of their users, particularly vulnerable populations like young people. Odgers (220) argues that age restrictions and mobile device bans may prove ineffective—or even counterproductive—considering what is known about adolescent behaviour. Regulation of the social media platforms to make them safer would be a more appropriate response.

Wilkinson and Pickett (175)’s work highlights the role of socio-economic inequality, emphasizing how growing disparities and economic stressors exacerbate mental health issues. Focusing on a deeper level, the economic doctrine of neoliberalism, which has dominated the political economy of the globe since the 1980s, can also be hypothesised to be a common force underpinning many of these trends in youth mental health and indeed wider effects on society (86, 221). Neoliberalism, with its promotion of individualism over collectivism and the common good, market deregulation, privatization, a weakened role and respect for government and even democracy, reduced government intervention erosion of the welfare state, creates an environment where young people face heightened economic pressures, social inequalities, and reduced and fragmented health and social safety nets (169, 222). These proposed contributory causes may (223) collectively contribute to the deteriorating trends in youth mental health, however their relative importance is yet to be determined scientifically. To explore whether youth mental health trends reflect surface manifestations of deeper underlying societal changes, new methodologies, including potentially illuminating cross-national comparisons could be employed (108, 224).

### 4.2 Methodologies required to improve clarity and guide public health and political and social measures

Further research is urgently required to understand megatrends impacting mental ill-health in order to quantify their relative effect and estimate their malleability. Subsequently the effective implementation of strategies that target such harmful forces undermining this and other related aspects of public health must be pursued. Firstly, prospective studies utilising comparable sampling methods and measurement approaches are needed to validate trends within and across specific regions and nations, including and especially need to include low- and middle-income countries. This includes using equivalent measures across repeated timepoints (225). A global focus is needed to coordinate national and harmonised surveillance efforts in monitoring trends (226). While the Global Burden of Disease studies provide essential descriptive epidemiology data on incidence, prevalence, mortality, morbidity and disability (227), large longitudinal studies of representative samples are needed to monitor trends and changes within cohorts by assessing young people’s psychosocial development prior to, during and following the developmental stage of emerging adulthood (225, 228). These studies should incorporate comparable measures of potential explanatory factors. By collecting data on a wide range of explanatory factors such as social media usage, economic precarity, and educational pressure, and performing sophisticated multivariate regression analyses, it may be possible to quantify their potential individual and combined effects on mental health outcomes (229).

Causality could be guided by criteria such as temporality, strength and consistency of association, and dose-response relation (230). How these variables interact, and their cumulative effects on mental health require exploration in addition to identifying protective factors. For example, path analysis (231, 232), systems dynamics (233), and structural equation modelling (SEM) (234, 235) model complex relationships between variables to identify direct and indirect pathways influencing mental health. Using advanced statistical software to construct SEM models that include multiple variables and pathways can provide a detailed understanding of how different factors interact to affect mental health (234). Additionally, big data and machine learning can analyze large datasets to identify patterns and predictors of mental health outcomes (236–238). Collecting and analyzing big data from sources such as social media, health records, and surveys, and using machine learning techniques, can uncover trends and predictive factors for youth mental health (237).

Conducting comprehensive literature searches, systematic reviews, and meta-analyses can summarize findings, assess the relative importance of different candidate causes, and identify overall trends and patterns (239). Qualitative research, including in-depth interviews, focus groups, and case studies, with young people, parents, and educators can be used to explore their lived experiences with economic insecurity, educational pressures, and other stressors, and how these impact young people’s mental health (240, 241).

A range of robust approaches (e.g., interrupted time series, cross-cohort comparisons) are needed that contribute to the triangulation of findings and increase confidence in inferring causality (242). This may guide the identification of new at-risk populations and can inform preventive and early intervention strategies where modifiable factors are targeted and their effect on trends are monitored (243, 244). Intervention studies can offer a starting point to implementing and evaluating interventions aimed at addressing identified risk factors and improving mental health outcomes (245, 246). Designing and testing interventions, such as school-based mental health programs or community support initiatives and measuring their effectiveness through stepped wedge, cluster and other forms of randomized controlled trials or other rigorous evaluation methods, can provide more robust evidence for effective strategies (247).

Improving the measurement of cross-sectoral impacts of mental health prevention and treatment costs requires innovative methodologies. By expanding the scope of economic evaluations to include broader cost perspectives and utilizing cost-benefit and return on investment analyses, decision makers can gain valuable insights that extend beyond the healthcare sector (14). Employing methods like Multiple Criteria Decision Analysis (MCDA) can help integrate these economic impacts with other relevant considerations, providing a comprehensive approach for informed decision-making across different government portfolios (248). Moreover, conducting policy analysis to identify key policies related to candidate causes and using statistical methods to assess their impact on youth mental health by comparing outcomes pre- and post-policy implementation, can provide valuable insights (249, 250). There is some scope for limited randomized trials, particularly stepped wedged designs as new policies are introduced sequentially across different communities (251). By integrating these methodologies, researchers can improve the clarity and robustness of their findings, providing a more comprehensive understanding of the relative contributions of different factors to trends in youth mental health. This will give political leaders the opportunity to move beyond opinion, ideology and superficial populist responses.

### 4.3 Solutions using the Mrazek and Haggerty (1994) spectrum of interventions for mental health

**Figure 3:**
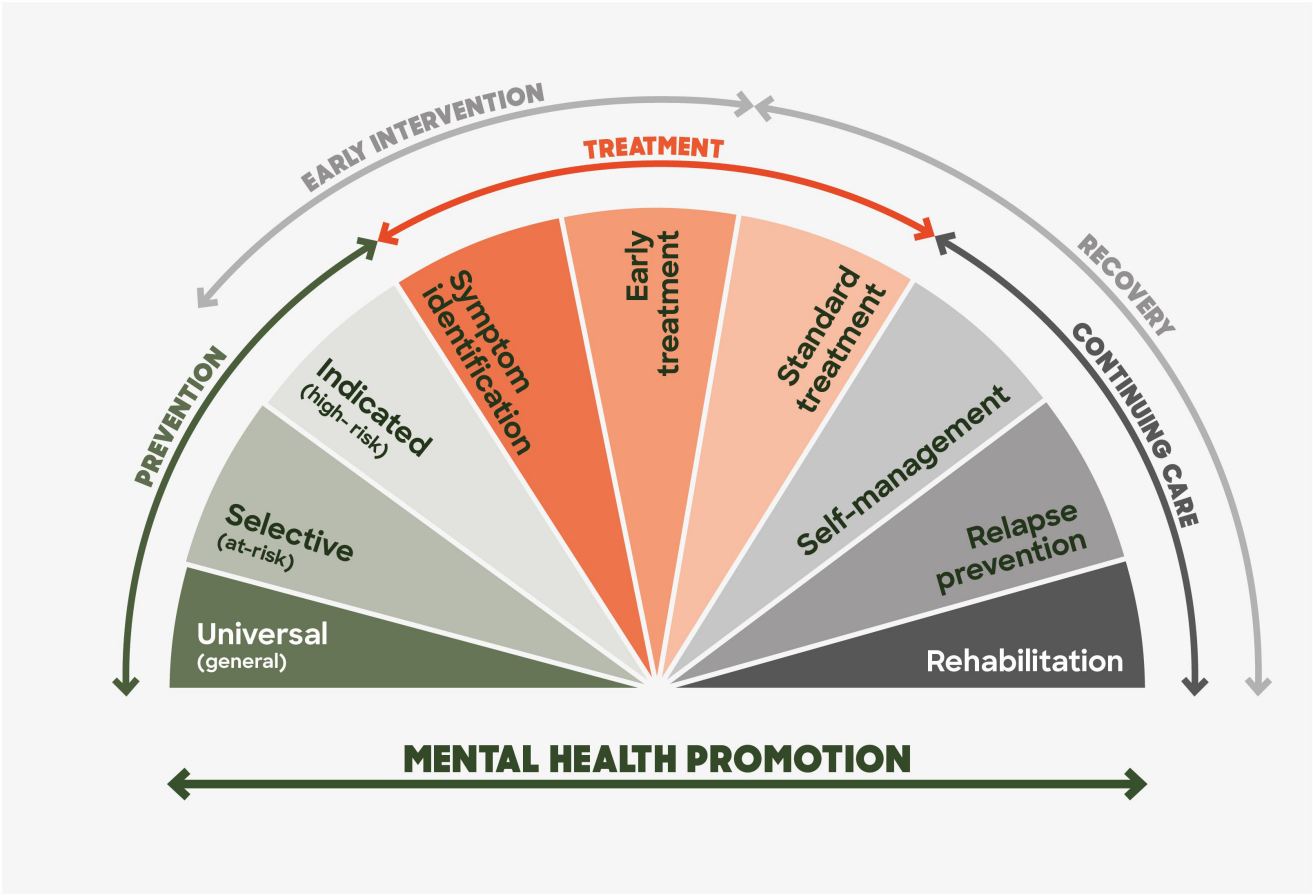
Model of the spectrum of interventions for mental health problems and mental health disorders: Adapted from Rickwood (84) and Mrazek & Haggerty (85).

Improving mental health outcomes requires a multifaceted approach that includes prevention, early intervention, and continuing care. This holistic approach is exemplified in the Mrazek and Haggerty model (85), which highlights prevention, early intervention, and sustained treatment and recovery support (See **Figure 3**).

#### 4.3.1 Prevention

Preventive measures aim to reduce the incidence and severity of mental health disorders by targeting megatrends as well as traditional risk and protective factors. Key players in this effort include communities, families, schools, and digital platforms. To effectively reduce the incidence and severity of mental health disorders, it is recommended to strengthen community support by fostering social connections and providing essential resources, which can act as protective factors against mental health issues (199, 252–254). Studies have shown a positive relationship between high-quality social relationships and subjective well-being across all ages (255). These findings indicate that the quality of relationships is more crucial than the quantity. However, having a large and diverse social network also provides protection against depression (255). High social support is important for adolescents who have experienced child maltreatment or other adversities and serves as a protective factor for LGBTQ adolescents (256–258).

Youth mentoring programs, where an adult mentor provides social support and engages in shared activities without offering psychotherapeutic interventions, have been used to reduce the risk of behavioural disorders among young people (259). Evidence suggests these programs can enhance social and emotional skills and lower the risk of behavioural disorders, substance use, and criminal offending (259, 260).

Schools should implement mental health programs that include early identification and intervention services, as these can significantly improve student outcomes and address mental health challenges (261). Additionally, leveraging digital platforms to offer accessible resources, psychoeducation, peer support, and connections to mental health professionals can reduce symptoms of anxiety and depression among young people (262). These strategies can be integrated across different levels of prevention:

Universal prevention targets broad risk factors, protective factors, and megatrends for an entire population without regard to individual risk factors. For example, integrating mental health education into school curricula helps to normalize discussions about mental health and provide students with tools for managing stress and emotional challenges (261, 263). This may also include school-based behavioral interventions to minimize risk of bullying and peer rejection (264) as well as substance abuse (265). Conversely, the literature also suggests that universal interventions may be ineffective and, in some cases, potentially harmful (266, 267). For example, a systematic review of studies conducted in UK mainstream schools found that school-based universal interventions targeting the promotion of emotional or mental wellbeing, or the prevention of mental health difficulties demonstrated neutral to small effects (268).

The identification of potent harmful megatrends strengthens the momentum for and emphasis upon both universal and selective preventive strategies (14). However, even if the relative contribution of these megatrends and new risk factors can be clarified, how malleable they will be is quite another question. It is likely to be not merely a scientific one but a political and economic one which, when posed, is likely to provoke controversy and resistance. It took forty years for neoliberalism to displace Keynsian economic theory and although there is an emerging case being mounted within the field of progressive economics for a reengineering and humanization of capitalism in the service of the common good (269, 270), this will be resisted and would in any case take time. One only needs to reflect on how “science” has been misused to oppose efforts to counter efforts to decrease smoking and exposure to asbestos, and to respond to the climate crisis to anticipate how “merchants of doubt” would be mobilized to whitewash and defend harmful policies which threaten vested interests (271).

Selective prevention targets on at-risk groups. Selective interventions are aimed at individuals who are at a significantly higher risk of developing a mental disorder compared to the general population, despite being asymptomatic. For instance, targeted interventions for LGBTIQA+ youth can address unique stressors and reduce the risk of mental health issues (272). One might even assert that a substantial proportion of young people are an at-risk segment of society and are already showing serious warning signs ahead of the remainder of society.

Indicated prevention transitions into early intervention, providing support for individuals showing early signs of mental health issues. Programs that identify, treat and support young people with emerging mental health problems can prevent the escalation of symptoms (90, 273–275). Research suggests that indicated interventions have successfully achieved their goals by strengthening service engagement, shortening the duration of untreated illness, and coordinating with secondary prevention efforts (273, 276). This is potentially a “sweet spot” for most potential health and economic gain and cost-effectiveness.

A balanced approach that integrates universal, selective, and indicated prevention research and interventions is essential for addressing the mental, physical and social needs of young people (277).

#### 4.3.2 Early intervention

Early intervention is considered a “best buy” in healthcare due to its cost-effectiveness and the substantial evidence supporting its benefits (274). Integrated and enhanced primary care youth services offer comprehensive support, addressing a range of mental health issues before they become severe (278). The “Integrated Behavioural Health in Primary Care” (IBHPC) model is an example, where behavioural health providers are embedded in primary care practices to offer immediate support and referrals (279). These services are designed to provide comprehensive, accessible, and coordinated care for young people, integrating mental health services into primary care settings. For instance, *headspace* serves as a model of Early Intervention, offering stigma-free, integrated support for mental health, substance use, and vocational needs while ensuring all help-seeking youth have access to care (14). This approach aims to address mental health issues early and effectively, leveraging the strengths of both primary care and specialized mental health services. For example, early psychosis intervention has been a focus for several decades, providing specialized care for young people experiencing their first episode of psychosis. This has now expanded to include transdiagnostic platforms of care, which address various mental health conditions within a unified framework (13).

#### 4.3.3 Treatment, continuing care and recovery support

Holistic, evidence-based treatment approaches are essential for sustained mental health and well-being. Once people remit with the benefit of early treatment it is crucial as with cancer to keep them well or also intervene early should relapse occurs. Recovery-oriented approaches recognize the importance of peer support networks and community resources (280). Group-based psychosocial interventions for children and adolescents exposed to traumatic events in humanitarian settings have been found to be effective in low- and middle-income countries. For example, Alzaghoul et al. (281) found that a skills-based trauma-focused CBT program significantly reduced PTSD and depression scores compared to waitlist groups. On the other hand, another study showed a small beneficial effect of psychosocial support on PTSD symptoms at four weeks post-intervention, but no difference in depressive and anxiety symptoms between treatment and control groups (282). Community resources, including support groups and local mental health services, play a vital role in continuing care. These resources help individuals maintain their mental health gains post-treatment and provide ongoing support to prevent relapse (283).

While there has been a real, yet modest and piecemeal, expansion of treatment availability and uptake for some young people, thanks to model innovation, the prevalence and impact of mental health conditions have not decreased in almost 25 years and are rising among young people (14). There is a need for a radical change and decisive action which will involve a major boost to prevention, early intervention and innovative youth focused treatment systems (14, 263, 284).

### 4.4 Barriers, strategies and opportunities, including economic and political considerations

Prevention, early diagnosis and intervention, and sustained access to evidence-based care have catalysed progress for other non-communicable diseases, leading to significant reductions in the prevalence and impact of conditions such as cardiovascular disease and some forms of cancer (285–287). These fundamental components of health care have not received the same recognition, urgency for deployment, and investment in mental health (288–290). This explains the lack of reduction in the prevalence and burden of mental disorders over the past 25 years (291). Worse still there has been a parallel rise in rates of youth mental ill-health, increased help-seeking and greater need for accessible and safe mental health services among young people (30, 201). However mental health services for young people continue to be all too poorly designed, sparse in coverage and depth, and under-resourced, even in countries that have led youth mental health reform (13). Contributing to these shortcomings are a range of personal and structural barriers. Young people may face personal barriers such as a lack of awareness about available services, mistrust of those services, stigma, misconceptions surrounding mental health, and fears around seeking help (292). Structural barriers include the absence of supportive initiatives within young people’s circles of support (such as family, community, and educational settings), limited-service capacity, and inadequate funding (292). Economic and political factors also present significant barriers to improving the mental health landscape for young people. Unaccountable government policies, harmful and hidden economic orthodoxy, and the impact of unrestrained and unregulated commercial and business activity can be extremely harmful and result in large cohorts of the population being exposed to iatrogenic effects and commercial drivers of mental ill health (293, 294). This is partially recognized but often overlooked in political and economic decision-making. There is a need for mental health impact analyses or statements (295), similar to environmental impact assessments, to be conducted before policies and projects are approved. Privatisation of health care and other economic barriers limit access to mental health services, exacerbate health inequity, and constitute frank discrimination against the mentally ill (296, 297). This scandal results in underfunded mental health programs and insufficient insurance coverage (298).

Strategic investment in both prevention, early intervention, and recovery and improved public health approaches could enable countries to achieve much greater improvements in mental health, save a lot of money and future proof their societies as a result. This includes increased preventive efforts targeting risk factors and determinants of mental illness (e.g., social, economic and environmental), as well as improved provision of evidence-based early intervention that has the capacity to meet the volume of demand (14). Additionally, as discussed in the previous sections, a range of methodological approaches should be utilized to understand the megatrends related to young people’s mental health.

Political will is essential for enacting policies that deliver mental health equity, such as increased funding for mental health research proportional to its share of the burden of disease and its impact on society, implementation of school-based mental health programs, and the establishment of accessible mental health services (294, 299). Political will is directly proportional in a democracy to the level of public activism and demand for reform, which needs to be energised and mobilised (300). Economic stability and growth can also indirectly impact mental health by reducing socio-economic adversity, one of the key contributors to mental health issues particularly among young people (68, 171, 175, 176). Policies aimed at reducing income inequality, improving education systems, and providing health and social safety nets can have positive effects on young people’s mental health (301, 302).

The mounting evidence of increasing youth mental ill-health combined with the persistent neglect of mental health care globally highlights the urgent need for increased action on prevention and early intervention of mental disorders. The scale of this crisis extends beyond the disorders that have been the main focus of this review, as other potentially serious mental illnesses that typically emerge during adolescence and young adulthood, such as eating disorders, have also increased in incidence (303). An immediate call to action was recently declared, with a whole of society approach invited to address growing rates of youth mental ill-health (18). To support this global endeavour, core principles to guide youth mental health care in low-, middle- and high-income countries are available (304) and a global blueprint to transform prevention and early intervention in youth mental health has been developed (305, 306). An urgent and serious public health response to the rising mental health needs of young people is required. This has the capacity to yield massive personal, social, and economic returns. If implemented effectively, major gains seen in other areas of health such as cancer and cardiovascular disease are also possible for youth mental health, and the mental health field and community globally.

## 5 Limitations

It is possible that factors not examined here, such as poor access to and quality of mental health care, have led to an increased prevalence, rather than incidence, of mental health issues among youth, as untreated or inadequately treated conditions may worsen and persist over time. Another limitation of this paper is the generalizability of findings. This lack of generalizability could be problematic as it overlooks some of the unique socio-economic, cultural, and environmental factors that influence youth mental health in diverse global contexts. Furthermore, trends and contributing factors in low- and middle-income countries remain unclear owing to the dominant focus on high-income countries, stemming from the fact that most data and literature comes from the latter. Another limitation of this review is the exclusion of neurodevelopmental disorders (NDDs), such as autism spectrum disorder (ASD) and attention-deficit/hyperactivity disorder (ADHD), from the primary analysis. While these conditions have seen a significant rise in diagnoses over the past century and an increase in diagnosis-seeking behaviour post-pandemic, their prevalence and trends remain uncertain due to the lack of robust community surveys. This gap in data also applies to evidence regarding the time trends of several youth mental health disorders, including bipolar disorder, schizophrenia, and eating disorders, which tend to be absent from many of the surveys of mental health. Future research should aim to address these gaps to provide a more comprehensive understanding of mental health trends.

## 6 Conclusion

The increasing evidence of deteriorating mental health among young people highlights a significant global public health crisis. The substantial rise in anxiety, psychological distress, and depression since the mid-1990s is alarming and deeply concerning. Factors such as climate change, family environment, educational pressures, socio-economic precarity, intergenerational inequality and the rise of social media contribute to this trend, though each only partially explains the increase and many of them may stem from a deeper malaise with political and economic roots. Methodological constraints and unexamined risk factors and trends further complicate our understanding. The COVID-19 pandemic is likely to have exacerbated the crisis but, in a way, fully accounts for it, emphasizing the urgent need for dynamic research, prevention and a new wave of innovation in youth mental health and social care. Facing these challenges can mitigate and reverse the harm, reduce the prevalence and impact of mental illness and deliver vital and essential benefits to our societies and economies globally.
