# Supplementary material for: The youth mental health crisis: analysis and solutions
Source: Front Psychiatry. 2025 Jan 21;15:1517533. doi: 10.3389/fpsyt.2024.1517533 (PMC11790661; doi:10.3389/fpsyt.2024.1517533)
Supplement: Supplementary file 1 [file Table1.docx]

Table 1: Trends in youth mental health relevant to anxiety, depression, internalising and emotion problems, somatic symptoms, suicidal thoughts/behaviours, schizophrenia, mood disorders, ED, psychological distress, and wellbeing

| **First author** | **Country or region** | **Study design (number of data time points)** | **Years studied (n)** | **Last year of assessment** | **Total years of follow-up** | **Population size (e.g., cohort size or averaged sample size per year)** | **Age, yrs** | **Measure** | **Findings** | **Mental disorder** | **Trends** **Increase 1, Decrease -1, Stable 0** |  |
| --- | --- | --- | --- | --- | --- | --- | --- | --- | --- | --- | --- | --- |
| Collishaw (1) | UK | Cross-sectional and longitudinal study (3) | 1974–1999 (18,660) | 1999 | 25 | 6220.0 | 15–16 | Rutter A Scale (1974 & 1986 data), SDQ (1999 data) (parent-report) | Emotional problems were stable (1974–86) before increasing (1986–99) (both genders; 10.5% to 16.9%). Estimated odds of a high emotional problem score in 1999 were 1.7 times greater than in 1986. | Emotional problems | 1 |  |
| Costello (2) | UK, England | Meta-analysis | 1965–1996^b^ birth yr (60000) | 1996 | 31 | 60000.0 | 13–18 | Structured diagnostic interviews (based on DSM or ICD) (self-, parent- or teacher-report) | No evidence of an increase in prevalence of depression. | Depression | 0 |  |
| Fichter (3) | Germany  Greece | Repeated cross-sectional survey, school-based (2) | 1980–1998 (5,551) | 1998 | 18 | 2775.5 | 13–19 | GHQ-28 (self-report) | Significant increase in anxiety, severe depression, and somatic complaints. Scores were higher for females compared to males. | Anxiety | 1 |  |
|  |  |  |  |  |  |  |  |  |  | Depression | 1 |  |
|  |  |  |  |  |  |  |  |  |  | Somatic symptoms | 1 |  |
| Jorm (4) | Australia | Repeated cross-sectional household survey (2) | 1995–2003/4^b^  (5471) | 2003 | 8 | 2735.5 | 20–24 | 4 Neurotic Symptoms questionnaire (self-report) | Psychological distress increased among males. | Distress | 1 |  |
| Tick (5) | Netherlands | Repeated cross-sectional study, population-based (2) | 1993–2003 (1,905) | 2003 | 10 | 952.5 | 11–18 | Youth Self-Report | Increase in thought problems, somatic complaints, and internalising problems (girls). | Emotional problems | 1 |  |
|  |  |  |  |  |  |  |  |  |  | Somatic symptoms | 1 |  |
| Tick (6) | Netherlands | Repeated cross-sectional study, population-based (3) | 1983–2003 (2,173) | 2003 | 20 | 724.3 | 12–16 | CBCL (parent-report), Teacher’s Report Forms | Small increase in anxious⁄depressed, somatic problems, and internalising scales (1993–2003). | Emotional problems | 1 |  |
|  |  |  |  |  |  |  |  |  |  | Somatic symptoms | 1 |  |
| Sigfusdottir (7) | Iceland | Repeated cross-sectional survey, school-based (4) | 1997–2006 (21,245) | 2006 | 9 | 5311.3 | 14–15 | Anxiety and depression dimension scales defined by Derogatis et al. 1971 (self-report) | Significant increase in anxiety (both genders) and depressive symptoms (girls only). | Anxiety | 1 |  |
|  |  |  |  |  |  |  |  |  |  | Depression | 1 |  |
| Levin (8) | Scotland | Repeated cross-sectional survey, school-based (4) | 1994–2006 (19,393) | 2006 | 12 | 4848.3 | 11–15 | Health Behaviour in School-aged Children Survey/Symptom Checklist (self-report) | Significant increase in all mental wellbeing measures for girls and in all but confidence among boys. Significant decrease in odds of internalising problems over time for both genders. | Low wellbeing | -1 |  |
|  |  |  |  |  |  |  |  |  |  | Internalising | -1 |  |
| Sweeting (9)  West (10) | West Scotland | Repeated cross-sectional survey, home and school-based (3) | 1987–2006 (5,895) | 2006 | 19 | 1965.0 | 15 | GHQ-12 (self-report) | Psychological distress caseness rates in 1987, 1999 and 2006 were 12.7%, 15.1% and 21.5% (males) and 18.8%, 32.5% and 44.1% (females). Marked increase in caseness among females (1987–1999) and both genders (1999–2006). | Distress | 1 |  |
| Calling (11) | Sweden | Longitudinal survey (4) | 1980/1–2004/5 (2,970) | 2004 | 24 | 742.5 | 16–23 | Item: ‘do you suffer from nervousness, uneasiness, or anxiety?’ (self-report) | Anxiety increased from 5.0% to 13.4% in males and from 11.8% to 31.0% in females. | Anxiety | 1 |  |
| Hagquist (12) | Sweden | Repeated cross-sectional survey, school-based (5) | 1985/6–2005/6 (12,368) | 2005 | 20 | 2473.6 | 13–15 | Health Behaviour in School-aged Children Symptom Checklist (self-report) | Significantly higher rates of internalising problems in 2005/2006 compared to 1985/1986 (especially for girls). | Internalising | 1 |  |
| Hagquist (13)^a^ | Sweden, Värmland | Repeated cross-sectional survey, school-based (6) | 1988–2005 (15,206) | 2005 | 17 | 2534.3 | 15–16 | Psychosomatic Problems scale (self-report) | Psychosomatic problems more than doubled (1988–2005; boys 5.0% to 10.6%; girls 16.7% to 28.9%). | Internalising | 1 |  |
| Kosidou (14) | Sweden, Stockholm | Repeated cross-sectional survey (5) | 1996/7–2004/5 (1,284) | 2004 | 8 | 256.8 | 16–24 | Item: ‘do you suffer from nervousness, uneasiness, or anxiety?’ (self-report) | For men, prevalence of anxiety increased from 10.9% (1997) to 18.8% (2003) and decreased to 17.1% (2005). Women showed a constant increase: 21.3% (1997) to 37.7% (2005). | Anxiety | 1 |  |
| Dey (15) | Switzerland | Repeated cross-sectional survey, school-based (4) | 1994–2006 (33,625) | 2006 | 12 | 8406.3 | 11–15 | Health Behaviour in School-aged Children Symptom Checklist (self-report) | Relative to 1994, lower levels of internalising problems experienced in 1998, 2002 and 2006 (changes were minor). Somatic complaints increased slightly but consistently from 1994 (mean score: 2.82) to 2006 (3.36). | Internalising | -1 |  |
|  |  |  |  |  |  |  |  |  |  | Somatic symptoms | 1 |  |
| Collishaw (16) | UK, England | Repeated cross-sectional survey, household-based (2) | 1986–2006 (7,847) | 2006 | 20 | 3923.5 | 16–17 | Malaise Inventory (self-report), GHQ-12 (self-report), Rutter A Scale (parent-report) | Increase in emotional problems (especially girls). Greatest changes seen for symptoms of worry, irritability, fatigue, sleep disturbance, panic and feeling worn out/under strain. | Emotional problems | 1 |  |
| Maughan (17) | UK | Repeated cross-sectional survey (2) | 1999–2004 (7,174) | 2004 | 5 | 3587.0 | 11–15 | SDQ (parent, teacher, and self-report) | Emotional problems: significant decrease (self-report). Total difficulties score: significant decrease (parent ratings). | Emotional problems | -1 |  |
| Twenge (18) | US | Cross-temporal meta-analysis | 1938–2007 (77,576) | 2007 | 69 | 77576.0 | High school/ college students | Minnesota Multiphasic Personality Inventory (self-report) | Each successive generation of high school and college students reported more mental health problems, including depression, even when sample gender composition, region and decreases in defensive responding were controlled. | Depression | 1 |  |
| Baxter (19) | Worldwide, 21 regions | Meta-analysis (3) | 1990–2010^b^ | 2010 | 20 | N/A | 15–24 | DSM or ICD criteria, GHQ | No increase in anxiety or major depressive disorder. Significant increase in psychological distress, including adolescent females. | Distress | 1 |  |
|  |  |  |  |  |  |  |  |  |  | Anxiety | 0 |  |
|  |  |  |  |  |  |  |  |  |  | Depression | 0 |  |
| Bor (20) | Worldwide, 12 nations | Systematic review | 1983–2010 (>100,000) | 2010 | 27 | N/A (>100,000) | 11–18 | Varied across studies | Increasing symptom burden (internalising problems), especially for girls. | Internalising | 1 |  |
| Bremberg (21) | Netherlands/ Nordic nations | Systematic review | 1990–2010^b^ | 2010 | 20 | N/A | 15–24 | Varied across studies | Slight increase in internalising mental health symptoms; increase was marked in Sweden. | Internalising | 1 |  |
| McMartin (22) | Canada | Population-based longitudinal survey (8) | 1994/5–2008/9 (20,409) | 2008 | 14 | 2551.1 | 12–15 | Survey items/behaviour scale based on  DSM-III (self-report) | Prevalence of depression/anxiety relatively stable for 12–13-year-olds; small significant decrease for 14–15-year-olds. | Depression | 0 |  |
|  |  |  |  |  |  |  |  |  |  | Anxiety | 0 |  |
| Markkula (23) | Chile | Repeated cross-sectional survey, household-based (2) | 2003–2010 (970) | 2010 | 7 | 485.0 | 18–25 | Composite International Diagnostic Interview, short form (self-report) | Stable prevalence of major depressive episode: 17.1% (2003) and 18.1% (2010). | Depression | 0 |  |
| Sourander (24) | Finland | Repeated cross-sectional survey, school-based (2) | 1998–2008 (3,027) | 2008 | 10 | 1513.5 | 13–17 | SDQ (self-report) | Substantial stability in emotional problems. | Emotional problems | 0 |  |
| Torikka (25) | Finland | Repeated cross-sectional survey, school-based (6) | 2000/1–2010/11 (618,084) | 2010 | 10 | 103014.0 | 14–16 | Beck Depression Inventory (12-item Finnish modification) (self-report) | Severe depression stable in females: 4% (2000/1) to 4.7% (2010/11), peaked 2010/11. Also stable in males: 2.1% (2000/1) and 2.2% (2010/11), peaked 2008/9. Mild/moderate depression: stable or decreased (both genders, 2000–2011). | Depression | 0 |  |
| von Soest (26) | Norway | Repeated cross-sectional survey, school-based (3) | 1992–2010 (9,245) | 2010 | 18 | 3081.7 | 16–17 | Depressive Mood Inventory (self-report) | 1992–2002: prevalence of high depressive symptoms increased significantly (both genders). 2002–2010: no significant changes (but symptoms increased for girls). | Depression | 1 |  |
| Konowałek (27) | Poland | Repeated cross-sectional survey, school-based (2) | 2000–2011 (444) | 2011 | 11 | 222.0 | 16 | Youth Self-Report | Increase in emotional/internalising problems (19.5% to 31.9%). No significant association of year of study and somatic complaints (but 2011 respondents more likely to be included in the clinical group). | Emotional problems | 1 |  |
|  |  |  |  |  |  |  |  |  |  | Somatic symptoms | 0 |  |
| Kozina (28) | Slovenia | Cross-sectional cohort study, school-based (2) | 2007–2011 (8,784) | 2011 | 4 | 4392.0 | 14 | Anxiety Scale for Children and Adolescents (self-report) | Small significant increase in anxiety (mean scores: 30.03 in 2007 and 33.55 in 2011). | Anxiety | 1 |  |
| van Geelen (29)^a^ | Sweden, Värmland | Repeated cross-sectional population-based study, school-based (8) | 1988–2011 (19,823) | 2011 | 23 | 2477.9 | 15–16 | Psychosomatic Problems scale (self-report) | Steady increase in psychosomatic symptoms (significant in 2005/2008 vs. 1988) even when accounting for functional impairment (girls significantly higher than boys). | Internalising | 1 |  |
| Ross (30) | UK, England | Household panel study (18) | 1991–2008 (6,212) | 2008 | 17 | 345.1 | 16–24 | GHQ-12 (self-report) | Small significant increase in mean scores of psychological distress (women); non-significant decrease for men. | Distress | 1 |  |
| Mehta (31) | US | Repeated cross-sectional survey (4) | 2005/6–2011/2 (3,135) | 2011 | 6 | 783.8 | 18–24 | PHQ-9 (self-report) | Prevalence of major depression increased from 1.87% (2005/6) to 3.47 (2011/2). | Depression | 1 |  |
| Collishaw (32) | Worldwide, 9 nations | Review | 1970–2012 (711,085) | 2012 | 42 | 711085.0 | 10–26 | Varied across studies | Increase in adolescent emotional problems over the past 30 years in high-income countries, particularly among girls. | Emotional problems | 1 |  |
| Harvey (33) | Australia | Repeated cross-sectional household survey (5) | 2001–2014^b^  (65,491) | 2014 | 13 | 13098.2 | 18–24 | K10 (self-report) | Slight non-significant decline in high/very high levels of distress. | Distress | -1 |  |
| Lawrence (34)   Sawyer (35) | Australia | Repeated cross-sectional household survey (2) | 1998–2013/4 (4,777) | 2013 | 15 | 2388.5 | 12–17 | DISC-IV (parent and self-report) | Modest increase in major depressive disorder (from 2.9% to 5.0%) (self-report). Increased for females (3.1% to 5.8%) and males (2.7% to 4.3%) (parent report). | Depression | 1 |  |
| Van Droogenbroeck (36) | Belgium | Repeated cross-sectional household survey (2) | 2008–2013 (1,433) | 2013 | 5 | 716.5 | 15–25 | SCL-90-Revised, GHQ-12 (self-report) | Prevalence of anxiety and depression significantly increased for females and, to a lesser extent, males. Psychological distress decreased in 2013 for males; no significant change for females. | Depression | 1 |  |
|  |  |  |  |  |  |  |  |  |  | Anxiety | 1 |  |
|  |  |  |  |  |  |  |  |  |  | Distress | 0 |  |
| Comeau (37) | Canada | Repeated cross-sectional survey (2) | 1983–2014 (3,807) | 2014 | 31 | 1903.5 | 12–16 | Checklist based on DSM-III criteria (parent and self-report) | Prevalence of any disorder was stable although emotional disorder increased (9.2% to 13.2%). | Emotional disorder | 1 |  |
| Wiens (38) | Canada | Repeated cross-sectional household survey (7) | 2000/1–2013/4 (51,714) | 2013 | 13 | 7387.7 | 12–19 | Composite International Diagnostic Interview, short form (self-report) | No significant change in prevalence of major depressive episodes (2000–2014). Prevalence of self-reported mood disorder diagnosis by a health professional increased (2003–2014). | Depression | 0 |  |
| Due (39) | Denmark | Repeated cross-sectional survey, school-based (7) | 1991–2014 (31,664) | 2014 | 23 | 4523.4 | 11, 15 | Health Behaviour in School-aged Children Symptom Checklist (self-report) | Increasing prevalence of daily emotional problems (5.6% to 10.0%), especially in high/middle occupational social classes. | Emotional problems | 1 |  |
| Mishina (40) | Finland | Repeated cross-sectional survey, school-based (3) | 1998–2014 (4,508) | 2014 | 16 | 1502.7 | 13–18 | SDQ (self-report) | Overall, relatively stable or improved self-perceived mental health, although prevalence of emotional problems increased substantially (females only 17.8% to 26.5%). | Emotional problems | 1 |  |
| Noorbala (41) | Iran | Repeated cross-sectional survey (2) | 1999–2015^b^  (36600) | 2015 | 16 | 18300.0 | 15–24 | GHQ-28 | Prevalence of probable mental disorder (incl. somatic, anxiety and depressive symptoms) relatively stable (17.6% in 1999 and 17.0% in 2015). | Internalising | 0 |  |
| Kachi (42) | Japan | Repeated cross-sectional household survey (3) | 2007–2013 (9,491) | 2013 | 6 | 3163.7 | 12–18 | K6 (self-report) | Prevalence of psychological distress decreased significantly from 2007 (10.7%) to 2013 (7.6%). | Distress | -1 |  |
| Duinhof (43) | Netherlands | Repeated cross-sectional survey, school-based (5) | 2003–2013 (29,352) | 2013 | 10 | 5870.4 | 11–16 | SDQ (self-report) | Stable trends (emotional problems). | Emotional problems | 0 |  |
| van Vuuren (44) | Netherlands, Amsterdam | Repeated cross-sectional survey, school-based (10) | 2004–2013 (56,159) | 2013 | 9 | 5615.9 | 13–14 | SDQ (self-report) | Emotional problems were stable overall, although some evidence of an increase in girls. | Emotional problems | 0 |  |
| Clark (45)   Fleming (46) | New Zealand | Repeated cross-sectional survey, school-based (3) | 2001–2012 (27,174) | 2012 | 11 | 9058.0 | 12–18 | SDQ, Reynolds Adolescent Depression Scale, Short Form (self-report) | Prevalence of clinically significant depressive symptoms stable, although a decrease (2007) was followed by a slight significant increase (2012). Slight significant increase in emotional symptoms (7.1% in 2007 to 10.5% in 2012). | Depression | 0 |  |
|  |  |  |  |  |  |  |  |  |  | Emotional problems | 1 |  |
| Fink (47) | UK, England | Repeated cross-sectional survey, school-based (2) | 2009–2014 (22,737) | 2014 | 5 | 11368.5 | 11–13 | SDQ (self-report) | Similar levels of mental health difficulties in 2009 and 2014 – with exception of increase in emotional problems in girls (13.1% to 20.3%) and a decrease in total difficulties in boys. | Emotional problems | 1 |  |
| Pitchforth (48) | UK, England, Scotland, Wales | Repeated cross-sectional national survey (20) | 1995–2014^b^ (140830) | 2014 | 19 | 7041.5 | 13–24 | SDQ Emotional difficulties, GHQ, Warwick-Edinburgh Mental Wellbeing Scores (parent and self-report) | Little change in scores for psychological distress and emotional wellbeing. | Distress | 0 |  |
|  |  |  |  |  |  |  |  |  |  | Wellbeing | 0 |  |
| Mojtabai (49) | US | Repeated cross-sectional household survey (10) | 2005–2014 (351,250) | 2014 | 9 | 35125.0 | 12–25 | Structured interview based on DSM-IV (self-report) | Significant increase in major depressive episodes from 8.7% (2005) to 11.3% (2014) in adolescents and from 8.8% to 9.6% in young adults. Findings significant for ages 12–20. | Depression | 1 |  |
| Twenge (50) | US | Time-lag national survey (25) | 1991–2015 (506,820) | 2015 | 24 | 20272.8 | 13–18 | Bentler Medical and Psychological Functioning Inventory depression scale (self-report) | Depressive symptoms increased (2010 and 2015), especially among females. | Depression | 1 |  |
| Twenge (51) | US | Time-lag national survey (6, 16) | 1982–2012 (70,838)  1985–2013 (~200,000pa) | 2013 | 31 | 11806.33 4427.38 | Grade 12, college students | American Freshman and Monitoring the Future survey items (self-report) | High school students in the 2010s reported more somatic symptoms of depression than those in the 1980s. Depression less likely in college students in the 2010s (vs. 1980s). | Depression | -1 |  |
|  |  |  |  |  |  |  |  |  |  | Somatic symptoms | 1 |  |
| Weinberger (52) | US | Repeated cross-sectional national survey (12) | 2005–2015^b^ (607520) | 2015 | 10 | 50626.7 | 12–25 | National Survey on Drug Use and Health (based on DSM-IV, National Comorbidity Survey Replication, and National Comorbidity Survey Adolescent) (self-report) | Significant increase in depression: 8.7% to 12.7% (12–17-year-olds) and 8.8% to 10.3% (18–25-year-olds). Increase significantly more rapid for 12–17-year-olds than older adults. | Depression | 1 |  |
| Cosma (53) | Europe/ North America, 36 nations | Repeated cross-sectional survey, school-based (5) | 2002–2018 (193,058) | 2018 | 16 | 38611.6 | 11–15 | Health Behaviour in School-aged Children Symptom Checklist, Cantril ladder (self-report) | Small significant increase in internalising problems. Trends varied across countries: clear decline in mental wellbeing in Northern and Western Europe and Canada. Girls showed a considerably higher risk of low mental wellbeing. | Internalising | 1 |  |
|  |  |  |  |  |  |  |  |  |  | Low wellbeing | 1 |  |
| Wiens (54) | Canada | Repeated cross-sectional household survey (8) | 2011–2018 (71,700) | 2018 | 7 | 8962.5 | 12–24 | Self-reported professional diagnosis | Prevalence of anxiety disorder significantly increased from 2011 (6.0%) to 2018 (12.9%). Largest rise observed among adolescent females. | Anxiety | 1 |  |
| Ren (55) | China | Analysis of the Global Burden of Disease study (2) | 1990–2017^b^ | 2017 | 27 | N/A | 15–24 | Diagnosis of major depressive disorder and dysthymia (DSM and ICD) | Decreasing prevalence of depression. | Depression | -1 |  |
| Thorisdottir (56) | Iceland | Repeated cross-sectional population-based survey, school-based (6) | 2006–2016 (42,943) | 2016 | 10 | 7157.2 | 14–15 | SCL-90 (depression and anxiety dimensions) (self-report) | Girls: significant increase in mean levels of depressive symptoms (2009–2010 and 2012–2016); anxiety symptoms increased significantly (2012–2016). Boys: depressive symptoms stable; anxiety symptoms decreased. High levels of depression increased by 6.8% for girls and 1.6% for boys. | Anxiety | 1 |  |
|  |  |  |  |  |  |  |  |  |  | Depression | 1 |  |
| Sutcliffe (57) | New Zealand | Repeated cross-sectional survey, school-based (4) | 2001–2019 (34,548) | 2019 | 18 | 8637.0 | ~13–17 | WHO-5 Wellbeing Index, Reynolds Adolescent Depression Scale (self-report) | Good wellbeing significantly declined from 76.0% (2012) to 69.1% (2019). Depression symptoms significantly increased from 13.0% (2012) to 22.8% (2019). | Depression | 1 |  |
|  |  |  |  |  |  |  |  |  |  | Wellbeing | -1 |  |
| Bobrowski (58) | Poland (Warsaw) | Repeated cross-sectional survey, school-based (4) | 2004–2016 (4,445) | 2016 | 12 | 1111.3 | 15 | Center for Epidemiologic Studies Depression Scale (shortened version), 1 item from the Health Related Quality of Life-4 (self-report) | Internalizing problems increased significantly from 10.1% (2008) to 16.2% (2016). Increased significantly for both girls and boys | Internalising | 1 |  |
| Coley (59) | US | Cross-sectional national survey, school-based (7) | 1989–2017 (350,048) | 2017 | 28 | 50007.0 | 13–17 | Monitoring the Future survey items (self-report) | Depressive symptoms significantly increased in 2014–2017 compared to 2010–2013 levels. Significant rising levels of depressive symptoms since 2013. | Depression | 1 |  |
| Duffy (60) | US | Cross-sectional national survey (10) | 2007–2018 (788,235) | 2018 | 11 | 78823.5 | 21.25 (mean)/18–22 | Self-reported overwhelming anxiety, PHQ-9, Generalized Anxiety Disorder-7, National College Health Assessment, Flourishing Scale (self-report) | Rates of depression and anxiety increased – relative percentage increases of 34% and 24%, respectively. Rates of severe depression and severe anxiety more than doubled; marked increase in severe depression began in 2013. Larger increases in depression among women than men. Low wellbeing increased (9.6% to 21.0%, 2012–2017/8). | Depression | 1 |  |
|  |  |  |  |  |  |  |  |  |  | Anxiety | 1 |  |
|  |  |  |  |  |  |  |  |  |  | Low wellbeing | 1 |  |
| Goodwin (61) | US | Cross-sectional national survey (11) | 2008–2018^b^ (439691) | 2018 | 10 | 39971.9 | 18–25 | Survey item measuring self-reported nervousness | Significant increase in anxiety: 7.97% (2008) to 14.66% (2018). Increase significantly more rapid than 26–49 age group. | Anxiety | 1 |  |
| Keyes (62) | US | Cross-sectional survey, school-based (28) | 1991–2018 (512,283) | 2018 | 27 | 18296.5 | 13–18 | Monitoring the Future survey items (self-report) | Upward trend in depressive symptoms from 2012, peaking in 2018. Females: depressive symptoms decreased (1991–2007), which then reversed. Males: relatively similar trends although less marked increase. | Depression | 1 |  |
| Lipson (63) | US | Cross-sectional survey, university-based (9) | 2007–2017 (155,026) | 2017 | 10 | 17225.1 | 18–22^c^ | PHQ-2 (adapted version) (self-report) | Depression increased from 24.8% (2009) to 29.9% (2016/7). | Depression | 1 |  |
| Lu (64) | US | Repeated cross-sectional survey (6) | 2011–2016 (95,856) | 2016 | 5 | 15976.0 | 12–17 | Structured interview based on DSM-IV (self-report) | Significant increase from 2011 to 2016 in prevalence of lifetime depression (12.8% vs. 18.1%), 12-month depression (8.3% vs. 12.9%), and 12-month depression with severe functional impairment (5.7% to 9.2%). | Depression | 1 |  |
| Lu (65) | US | Repeated cross-sectional survey (9) | 2011–2019 (207,895) | 2019 | 8 | 23099.4 | 18–25 | Structured interview based on DSM-IV (self-report) | Significant increase in prevalence of 12-month major depressive episode (8.0% in 2011 to 13.0% in 2019) | Depression | 1 |  |
| Parks (66) | US, Minnesota, | Repeated cross-sectional survey, school-based (2) | 2016, 2019 (215,436) | 2019 | 3 | 107718.0 | 12–19 | PHQ-2 | Prevalence of depressive symptoms stable: 21.6% (2016) to 22.0% (2019) | Depression | 1 |  |
| Parodi (67) | US, Dane County | Repeated cross-sectional survey, school-based (3) | 2012–2018 (44,351) | 2018 | 6 | 14783.7 | 14–18 | Generalized Anxiety Disorder-2 (modified version) (self-report) | Prevalence of anxiety significantly increased from 34.1% (2012) to 44.0% (2018), especially among females | Anxiety | 1 |  |
| Twenge (68) | US | Repeated cross-sectional national survey (13) | 2005–2017 (212,913 adolescents) | 2017 | 12 | 16378.7 | 12–25 | Structured interview (adapted from the National Comorbidity Survey Replication), K6 (self-report) | Major depressive episode increased by 52% from 2005 (8.7%) to 2017 (13.2%) among 12–17-year-olds and by 63% from 2009 (8.1%) to 2017 (13.2%) among 18–25-year-olds. Serious psychological distress increased among 18–25-year-olds between 2008–2017 (7.7% vs. 13.1%). Increases larger among women. | Depression | 1 |  |
|  |  |  |  |  |  |  |  |  |  | Distress | 1 |  |
| Twenge (50) | US | Time-lag national birth cohort survey (26) | 1991–2016 (1.1m) | 2016 | 25 | 42307.7 | Grades 8, 10, 12 | Rosenberg Self-Esteem scale and survey items measuring happiness and self/life satisfaction (self-report) | Psychological wellbeing decreased noticeably in adolescents (2012–2016). | Low wellbeing | 1 |  |
| Wang (69) | US | Repeated cross-sectional survey, school-based (5) | 2009–2017 (75,613) | 2017 | 8 | 15122.6 | Grades 9–12 | Youth Risk Behavior Survey (self-report) | Psychological distress increased: 31.9% (2009) to 35.7% (2017). Depressive symptoms increased: 26.1% (2009) to 31.5% (2017). | Distress | 1 |  |
|  |  |  |  |  |  |  |  |  |  | Depression | 1 |  |
| Buli (70) | Sweden | Repeated cross-sectional surveys (5) | 2002-2018 (9196) | 2018 | 16 | 1839.2 | 15 | Health Behavior in School-aged Children (HBSC) symptom checklist. | Girls had significantly higher mean PSS scores than boys across all years. After 2010, PSS scores began to increase significantly for both girls (F=16.783, df=4, p<.001) and boys (F=8.157, df=4, p<.001), with a significant interaction term between sex and the year of survey (B=0.029; p=.026) indicating that the increase was more pronounced among girls. | Psychosis | 1 |  |
| Ahn-Horst (71) | United States | Cross-sectional analysis  of nationally representative data (13) | 2006-2019 (1.1b) | 2019 | 13 | 84615384.6 | 18-24 | National Ambulatory Medical Care  Survey (NAMCS | Between 2006 and 2019, there were an estimated 1.1 billion outpatient visits by adolescents and young adults, with 145 million (13.1%) involving a mental health condition. Mental health-related diagnoses were more prevalent among male patients (16.8%) compared to female patients (10.9%) (P < .001). | Low mental wellbeing | 1 |  |
| Baumgarten (72) | Germany | Two-waves of German Health Interview and Examination Survey (2) | 2003-2006 (6725); 2014-2017 (6145) | 2017 | 14 | 6435.0 | 11-17 | Self-report version of the Strength and Difficulties Questionnaire (SDQ) | Compared to boys, the SDQ total difficulties score for girls increased with age, with the mean value for 16-year-old girls rising from 3.37 to 3.85 between KiGGS baseline and wave 2 (boys: 2.07 to 2.08). Boys showed more mental health problems in most age groups according to parent-reported SDQ, while girls had higher problem scores in the SDQ self-report. | Emotional problems | 1 |  |
| McGorry (73) | Australia | Sequential Multiple Assignment Randomized Trial | 2016-2019 (342) | 2019 | 3 | 342.0 | 12–25 | Standardized clinical assessment and the Prodromal Questionnaire-16 (PQ-16) | Adding sequentially more specialized psychosocial and antidepressant treatments for individuals who did not achieve remission did not  result in improved outcomes. Low remission and high relapse rates. | Psychosis | 1 |  |
| Burke (74) | Australia | Secondary data analysis | 2012-2016 (1123) | 2016 | 4 | 1123.0 | 15-25 | Comprehensive Assessment of At-Risk Mental State (CAARMS) instrument | Out of 1138 young people with a first episode of psychosis (FEP), 13.7% attended an At-Risk Mental State (ARMS) clinic, and  7.6% attended other youth mental health services. Transitioning from an ARMS clinic, personality disorder clinic, or primary care  significantly reduced hospital admissions compared to those presenting directly with FEP. | Psychosis | -1 |  |
| AIHW (75, 76); Australian Bureau of Statistics (77) | Australia | Repeated cross-sectional household survey (5) | 1997–2020/1 (57,364)^c^ | 2020 | 23 | 11472.8 | 16–24 | K10 (self-report), World Mental Health – Composite International Diagnostic Interview | Anxiety disorder: increased from 15.4% (2007) to 31.5% (2020/1). Affective disorder: increased from 6.3% (2007) to 13.6% (2020/1).  Psychological distress (18–24-year-olds): in females, high/very high distress increased from 12.5% (1997) to 22.1% (2001) and decreased to 18.7% (2004/5) and 12% (2007). In males, rates increased from 6.8% (1997) to 10.8% (2001) to 12.4% (2004/5) and decreased to 7% (2007). | Distress | 0 |  |
|  |  |  |  |  |  |  |  |  |  | Anxiety | 1 |  |
|  |  |  |  |  |  |  |  |  |  | Affective disorder | 1 |  |
| Jensen (78) | Denmark | Repeated cross-sectional survey (4) | 2010–2021 (72,816) | 2021 | 11 | 18204.0 | 16–24 | 12-Item Short Form Health Survey version 2 (self-report) | Proportion scoring low on the mental health scale increased from 15.8% (2010) to 34.4% (2021) for females, and from 8.3% (2010) to 21.2% (2021) for males. | Depression | 1 |  |
|  |  |  |  |  |  |  |  |  |  |  |  |  |
| Thorisdottir (79) | Iceland | Longitudinal, population-based survey, school-based (3) | 2016–2020 (59,701) | 2020 | 4 | 19900.3 | 13–18 | SCL-90 (depression dimension), Short Warwick Edinburgh Mental Wellbeing Scale (self-report) | Significantly higher depressive symptoms in 2020 (mean: 20.30) than 2016 (17.96) and 2018 (18.54). Mental wellbeing significantly worsened (mean scores: 23.29 in 2020, 24.62 in 2018, and 25.04 in 2016). Outcomes significantly worse in girls. | Depression | 1 |  |
|  |  |  |  |  |  |  |  |  |  | Low mental wellbeing | 1 |  |
| Cerniglia (80) | Italy | Longitudinal survey, school-based (2) | 2019–2020 (813) | 2020 | 1 | 406.5 | 14-15 | Youth Self-Report | Increase in emotional/internalising problems and somatic complaints (boys and girls). | Emotional problems | 1 |  |
|  |  |  |  |  |  |  |  |  |  | Somatic symptoms | 1 |  |
| van der Velden (81) | Netherlands | Longitudinal cohort study (3) | 2012–2020 (850) | 2020 | 8 | 283.3 | 16–20 | 5-item Mental Health Inventory (MHI-5) | Moderate anxiety and depression were more prevalent in 2020 than in 2012 and 2016, but mean score differences were minimal or absent. | Anxiety | 0 |  |
|  |  |  |  |  |  |  |  |  |  | Depression | 0 |  |
| Burdzovic Andreas (82) | Norway | Accelerated longitudinal study, school-based (2) | 2018/9–2020 (2,536) | 2020 | 2 | 1268.0 | 16 | PHQ-9, adolescent version (self-report) | No significant differences in depression symptoms between the pre-COVID and COVID groups. Adolescents with elevated pandemic-related anxiety significantly more likely to have clinical-level depression symptoms than pre-COVID group. | Depression | 0 |  |
| Hafstad (83) | Norway | Longitudinal survey, school-based (2) | 2019–2020 (3,572) | 2020 | 1 | 1786.0 | 12–16 | Hopkins Symptom Checklist-10 (self-report) | Small significant increase in clinical levels of anxiety and depression (5.5% to 6.3%) that was driven by increase in age rather than COVID-19 pandemic. Mean scores higher for girls. | Anxiety | 1 |  |
|  |  |  |  |  |  |  |  |  |  | Depression | 1 |  |
| Gagné (84) | UK | Household panel study (33) | 1991–2018 (5,500–38,313 households)   2018/9–2020 (42,330) | 2020 | 29 | 2443.7 | 16–24 | GHQ-12 (self-report) | From 1991–2018, prevalence of cases (mental distress) increased from 13.8% to 19.2% among males and from 22.1% to 31.6% among females, pronounced increase in 2015–16 and between 2006–10 and 2015–16. Severe distress increased at a steeper rate (3.1% to 10.6% in men; 7.2 to 16.3% in women). April 2020: significant increase in risk of distress, but this decreased between April and July–September 2020. | Distress | 1 |  |
| Niedzwiedz (85) | UK | Longitudinal household panel study (4) | 2015/7––2020 (8,575) | 2020 | 5 | 2143.8 | 18–24 | GHQ-12 (self-report) | Psychological distress substantially increased during COVID-19 pandemic (19.4% in 2017–2019 to 30.6% in 2020, with younger people (18–24 years) experiencing the greatest relative increase. | Distress | 1 |  |
| Pierce (86) | UK | Longitudinal cohort study, household panel survey (6) | 2014/5–2020 (1,543) | 2020 | 6 | 257.2 | 16–24 | GHQ-12 (self-report) | Mean score (mental distress) increased from 10.9 (2014/5) to 14.7 (2020). Proportion with clinically significant level of mental distress increased from 19.8% (2014/5) to 36.7% (2020). Steep increase in 2020 particularly in young women. | Distress | 1 |  |
| Hawes (87) | US, New York | Longitudinal study (2) | 2014/9–2020 (451) | 2020 | 6 | 225.5 | 12–22 | Children’s Depression Inventory, Screen for Child Anxiety Related Symptoms (self-report) | Increased generalized anxiety and social anxiety symptoms independent of age. Increased depression and panic/somatic symptoms in females. | Anxiety | 1 |  |
|  |  |  |  |  |  |  |  |  |  | Depression | 1 |  |
|  |  |  |  |  |  |  |  |  |  | Somatic symptoms | 1 |  |
| Guo (88) | Taiwan | Analysis of medical records | 2019-2022 | 2022 | 3 | 340.0 | 18-Sep | International Classification of Diseases, tenth revision   (ICD-10 | The proportion of psychological fragility increased from 0.28% pre-pandemic to 0.4% during the pandemic and 0.8% post-pandemic. Suicide attempts, particularly involving overdose and injury, were highest post-pandemic at 0.42%, with ICU admission rates. | Suicidal behaviours | 1 |  |
| Mitiku (89) | Ethiopia | Systematic review | 1995-2023c | 2023 | 28 | N/A | 15-29 | Varied across studies | The prevalence of mental illnesses among Ethiopian children was 24.68%. Factors significantly associated with childhood mental illness included previous history of child abuse (OR: 5.65; 95% CI: 4.32-7.39), poor socio-economic status (OR: 1.94; 95% CI: 1.24-3.04), urban residence (OR: 1.93; 95% CI: 1.39-2.67), and being male (OR: 1.65; 95% CI: 1.15-2.38). | Low mental wellbeing | 1 |  |
| Matsumoto (90) | Japan | Time-series analysis | 2017-2021 (4228) | 2021 | 4 | 1057.0 | 18-Sep | ICD-10   (International Classification of Diseases, Tenth Revision) | There were 362 new diagnoses of eating disorders, 1,104 of schizophrenia, 926 of mood disorders, and 1,836 of somatoform disorders. Post-pandemic, the monthly rate of new diagnoses increased significantly for all these mental disorders: eating disorders (slope change 1.05, 95% CI 1.00-1.11), schizophrenia (1.04, 95% CI 1.01-1.07), mood disorders (1.04, 95% CI 1.01-1.07), and somatoform disorders (1.04, 95% CI 1.02-1.07). | ED | 1 |  |
|  |  |  |  |  |  |  |  |  |  | Schizophrenia | 1 |  |
|  |  |  |  |  |  |  |  |  |  | Mood disorders | 1 |  |
|  |  |  |  |  |  |  |  |  |  | Somatic symptoms | 1 |  |
| Prichett (91) | United States | Cross-sectional cohort   study using outpatient EMR encounter data | 2015-2022 (29117) | 2022 | 7 | 4159.6 | 20-Aug | ICD-10 | After COVID-19 began, depression and anxiety rates increased significantly across racial and gender subgroups, particularly among Hispanic and Asian females. Suicide risk-related diagnoses also notably rose among all female subgroups, with the largest increases observed in Asian females and Black females. | Depression | 1 |  |
|  |  |  |  |  |  |  |  |  |  | Anxiety | 1 |  |
|  |  |  |  |  |  |  |  |  |  | Suicidal thoughts/behaviours | 1 |  |
| Reyes (92) | United States | Cross-sectional (2) | 1999-2021 (No. of deaths= 5109) | 2021 | 22 | 2554.5 | 5-24 | ICD-10-CM   Mortality data from CDC WONDER | Between 1999 and 2021, 4,747 Asian American Pacific Islander (AAPI) youth in the USA died by suicide, with rates doubling from 3.6 to 7.1 per 100,000. Suffocation, firearms, and poisoning were the most common methods. Rates were higher among Asian American (AA) males, while more AA females reported depressive symptoms, including suicidal planning and attempts. | Suicidal thoughts/behaviours | 1 |  |
| Wong (93) | Hong Kong | Territory-wide household-based epidemiological study | 2019-2022 (3460) | 2022 | 3 | 3460.0 | 15-24 | Composite International Diagnostic Interview Screening Scale | 16.6% of individuals had any mental disorder, with 13.7% experiencing a 12-month Major Depressive Episode (MDE). MDE prevalence peaked at 18.1% during mid-2020 but varied across periods, influenced by different stressors like social unrest and COVID-19. | Depression | 1 |  |

^a^ Samples drawn from the same survey (Hagquist 2009 and van Geelen et al 2016). ^b^ Not reported for 12–25 age range. ^c^ 66.8% of sample aged between 18 and 22 (Lipson et al 2019); includes >24 years of age (AIHW, 2007; 2011, Mitiku, 2024)

References

1. Collishaw S, Maughan B, Goodman R, Pickles A. Time trends in adolescent mental health. Journal of Child Psychology and Psychiatry. 2004;45(8):1350-62.

2. Costello EJ, Erkanli A, Angold A. Is there an epidemic of child or adolescent depression? Journal of Child Psychology and Psychiatry. 2006;47(12):1263-71.

3. Fichter MM, Xepapadakos F, Quadflieg N, Georgopoulou E, Fthenakis WE. A comparative study of psychopathology in Greek adolescents in Germany and in Greece in 1980 and 1998—18 years apart. Eur Arch Psychiatry Clin Neurosci. 2004;254(1):27-35.

4. Jorm AF, Butterworth P. Changes in psychological distress in Australia over an 8-year period: evidence for worsening in young men. Aust N Z J Psychiatry. 2006;40(1):47-50.

5. Tick NT, Van der Ende J, Verhulst FC. Ten-year trends in self-reported emotional and behavioral problems of Dutch adolescents. Soc Psychiatry Psychiatr Epidemiol. 2008;43(5):349-55.

6. Tick NT, Van Der Ende J, Verhulst FC. Twenty-year trends in emotional and behavioral problems in Dutch children in a changing society. Acta Psychiatr Scand. 2007;116(6):473-82.

7. Sigfusdottir ID, Asgeirsdottir BB, Sigurdsson JF, Gudjonsson GH. Trends in depressive symptoms, anxiety symptoms and visits to healthcare specialists: a national study among Icelandic adolescents. Scandinavian Journal of Public Health. 2008;36(4):361-8.

8. Levin KA, Currie C, Muldoon J. Mental well-being and subjective health of 11- to 15-year-old boys and girls in Scotland, 1994-2006. Eur J Public Health. 2009;19(6):605-10.

9. Sweeting H, Young R, West P. GHQ increases among Scottish 15 year olds 1987-2006. Soc Psychiatry Psychiatr Epidemiol. 2009;44(7):579-86.

10. West P, Sweeting H. Fifteen, female and stressed: changing patterns of psychological distress over time. Journal of Child Psychology and Psychiatry. 2003;44(3):399-411.

11. Calling S, Midlov P, Johansson SE, Sundquist K, Sundquist J. Longitudinal trends in self-reported anxiety. Effects of age and birth cohort during 25 years. BMC Psychiatry. 2017;17:119.

12. Hagquist C. Discrepant trends in mental health complaints among younger and older adolescents in Sweden: an analysis of WHO Data 1985-2005. J Adolesc Health. 2010;46(3):258-64.

13. Hagquist C. Psychosomatic health problems among adolescents in Sweden-are the time trends gender related. Eur J Public Health. 2009;19(3):331-6.

14. Kosidou K, Magnusson C, Mittendorfer-Rutz E, Hallqvist J, Hellner Gumpert C, Idrizbegovic S, et al. Recent time trends in levels of self-reported anxiety, mental health service use and suicidal behaviour in Stockholm. Acta Psychiatr Scand. 2010;122(1):47-55.

15. Dey M, Jorm AF, Mackinnon AJ. Cross-sectional time trends in psychological and somatic health complaints among adolescents: a structural equation modelling analysis of 'Health Behaviour in School-aged Children' data from Switzerland. Soc Psychiatry Psychiatr Epidemiol. 2015;50(8):1189-98.

16. Collishaw S, Maughan B, Natarajan L, Pickles A. Trends in adolescent emotional problems in England: a comparison of two national cohorts twenty years apart. Journal of Child Psychology and Psychiatry. 2010;51(8):885-94.

17. Maughan B, Collishaw S, Meltzer H, Goodman R. Recent trends in UK child and adolescent mental health. Soc Psychiatry Psychiatr Epidemiol. 2008;43(4):305-10.

18. Twenge JM, Gentile B, DeWall C, Ma D, Lacefield K, Schurtz DR. Birth cohort increases in psychopathology among young Americans, 1938-2007: a cross-temporal meta-analysis of the MMPI. Clin Psychol Rev. 2010;30(2):145-54.

19. Baxter AJ, Scott KM, Ferrari AJ, Norman RE, Vos T, Whiteford HA. Challenging the myth of an "epidemic" of common mental disorders: trends in the global prevalence of anxiety and depression between 1990 and 2010. Depress Anxiety. 2014;31(6):506-16.

20. Bor W, Dean AJ, Najman J, Hayatbakhsh R. Are child and adolescent mental health problems increasing in the 21st century? A systematic review. Aust N Z J Psychiatry. 2014;48(7):606-16.

21. Bremberg S. Mental health problems are rising more in Swedish adolescents than in other Nordic countries and the Netherlands. Acta Paediatr. 2015;104(10):997-1004.

22. McMartin SE, Kingsbury M, Dykxhoorn J, Colman I. Time trends in symptoms of mental illness in children and adolescents in Canada. Can Med Assoc J. 2014;186(18):E672-E8.

23. Markkula N, Zitko P, Peña S, Margozzini P, Retamal C P. Prevalence, trends, correlates and treatment of depression in Chile in 2003 to 2010. Soc Psychiatry Psychiatr Epidemiol. 2017;52(4):399-409.

24. Sourander A, Koskelainen M, Niemela S, Rihko M, Ristkari T, Lindroos J. Changes in adolescents mental health and use of alcohol and tobacco: a 10-year time-trend study of Finnish adolescents. Eur Child Adolesc Psychiatry. 2012;21(12):665-71.

25. Torikka A, Kaltiala-Heino R, Rimpelä A, Marttunen M, Luukkaala T, Rimpelä M. Self-reported depression is increasing among socio-economically disadvantaged adolescents - repeated cross-sectional surveys from Finland from 2000 to 2011. BMC Public Health. 2014;14(1):408.

26. von Soest T, Wichstrom L. Secular trends in depressive symptoms among Norwegian adolescents from 1992 to 2010. J Abnorm Child Psychol. 2014;42(3):403-15.

27. Konowałek Ł, Wolanczyk T. Changes in emotional and behavioral problems between 2000 and 2011 among 16-year-old Polish children: a cross-sectional study. Child Psychiatry Hum Dev. 2018;49(5):757-65.

28. Kozina A. Developmental and time-related trends of anxiety from childhood to early adolescence: two-wave cohort study. Eur J Dev Psychol. 2014;11(5):546-59.

29. van Geelen SM, Hagquist C. Are the time trends in adolescent psychosomatic problems related to functional impairment in daily life? A 23-year study among 20,000 15-16 year olds in Sweden. J Psychosom Res. 2016;87:50-6.

30. Ross A, Kelly Y, Sacker A. Time trends in mental well-being: the polarisation of young people’s psychological distress. Soc Psychiatry Psychiatr Epidemiol. 2017;52(9):1147-58.

31. Mehta K, Kramer H, Durazo-Arvizu R, Cao G, Tong L, Rao M. Depression in the US population during the time periods surrounding the great recession. J Clin Psychiatry. 2015;76(4):e499-504.

32. Collishaw S. Annual research review: secular trends in child and adolescent mental health. Journal of Child Psychology and Psychiatry. 2015;56(3):370-93.

33. Harvey SB, Deady M, Wang MJ, Mykletun A, Butterworth P, Christensen H, et al. Is the prevalence of mental illness increasing in Australia? Evidence from national health surveys and administrative data, 2001-2014. Med J Aust. 2017;206(11):490-3.

34. Lawrence D, Johnson S, Hafekost J, Boterhoven De Haan K, Sawyer M, Ainley J, et al. The mental health of children and adolescents. Report on the second Australian Child and Adolescent Survey of Mental Health and Wellbeing. Canberra; 2015.

35. Sawyer MG, Reece CE, Sawyer ACP, Johnson SE, Lawrence D. Has the prevalence of child and adolescent mental disorders in Australia changed between 1998 and 2013 to 2014? J Am Acad Child Adolesc Psychiatry. 2018;57(5):343-50.

36. Van Droogenbroeck F, Spruyt B, Keppens G. Gender differences in mental health problems among adolescents and the role of social support: results from the Belgian health interview surveys 2008 and 2013. BMC Psychiatry. 2018;18(1):6.

37. Comeau J, Georgiades K, Duncan L, Wang L, Boyle MH. Changes in the prevalence of child and youth mental disorders and perceived need for professional help between 1983 and 2014: evidence from the Ontario Child Health Study. Canadian Journal of Psychiatry. 2019;64(4):256-64.

38. Wiens K, Williams JVA, Lavorato DH, Duffy A, Pringsheim TM, Sajobi TT, et al. Is the prevalence of major depression increasing in the Canadian adolescent population? Assessing trends from 2000 to 2014. J Affect Disord. 2017;210:22-6.

39. Due P, Damsgaard MT, Madsen KR, Nielsen L, Rayce SB, Holstein BE. Increasing prevalence of emotional symptoms in higher socioeconomic strata: trend study among Danish schoolchildren 1991–2014. Scandinavian Journal of Public Health. 2019;47(7):690-4.

40. Mishina K, Tiiri E, Lempinen L, Sillanmäki L, Kronström K, Sourander A. Time trends of Finnish adolescents’ mental health and use of alcohol and cigarettes from 1998 to 2014. Eur Child Adolesc Psychiatry. 2018;27(12):1633-43.

41. Noorbala AA, Bagheri Yazdi SA, Faghihzadeh S, Kamali K, Faghihzadeh E, Hajebi A, et al. Trends of mental health status in Iranian population aged 15 and above between 1999 and 2015. Arch Iran Med. 2017;20(11):S2-S6.

42. Kachi Y, Abe A, Ando E, Kawada T. Socioeconomic disparities in psychological distress in a nationally representative sample of Japanese adolescents: a time trend study. Aust N Z J Psychiatry. 2017;51(3):278-86.

43. Duinhof EL, Stevens GWJM, van Dorsselaer S, Monshouwer K, Vollebergh WAM. Ten-year trends in adolescents' self-reported emotional and behavioral problems in the Netherlands. Eur Child Adolesc Psychiatry. 2015;24(9):1119-28.

44. van Vuuren CL, Uitenbroek DG, Van Der Wal MF, Chinapaw MJM. Sociodemographic differences in 10-year time trends of emotional and behavioural problems among adolescents attending secondary schools in amsterdam, the netherlands. Eur Child Adolesc Psychiatry. 2018;27(12):1621-31.

45. Clark T, Fleming T, Bullen P, Crengle S, Denny S, Dyson B, et al. Health and well-being of secondary school students in New Zealand: trends between 2001, 2007 and 2012. J Paediatr Child Health. 2013;49(11):925-34.

46. Fleming TM, Clark T, Denny S, Bullen P, Crengle S, Peiris-John R, et al. Stability and change in the mental health of New Zealand secondary school students 2007-2012: results from the national adolescent health surveys. Aust N Z J Psychiatry. 2014;48(5):472-80.

47. Fink E, Patalay P, Sharpe H, Holley S, Deighton J, Wolpert M. Mental health difficulties in early adolescence: a comparison of two cross-sectional studies in England from 2009 to 2014. J Adolesc Health. 2015;56(5):502-7.

48. Pitchforth J, Fahy K, Ford T, Wolpert M, Viner RM, Hargreaves DS. Mental health and well-being trends among children and young people in the UK, 1995-2014: analysis of repeated cross-sectional national health surveys. Psychol Med. 2019;49(8):1275-85.

49. Mojtabai R, Olfson M, Han B. National trends in the prevalence and treatment of depression in adolescents and young adults. Pediatrics. 2016;138(6):e20161878.

50. Twenge JM, Joiner TE, Rogers ML, Martin GN. Increases in depressive symptoms, suicide-related outcomes, and suicide rates among U.S. adolescents after 2010 and links to increased new media screen time. Clinical Psychological Science. 2018;6(1):3-17.

51. Twenge JM. Time period and birth cohort differences in depressive symptoms in the U.S., 1982–2013. Social Indicators Research. 2015;121(2):437-54.

52. Weinberger AH, Gbedemah M, Martinez AM, Nash D, Galea S, Goodwin RD. Trends in depression prevalence in the USA from 2005 to 2015: widening disparities in vulnerable groups. Psychol Med. 2018;48(8):1308-15.

53. Cosma A, Stevens G, Martin G, Duinhof EL, Walsh SD, Garcia-Moya I, et al. Cross-national time trends in adolescent mental well-being from 2002 to 2018 and the explanatory role of schoolwork pressure. J Adolesc Health. 2020;66(S6):S50-S8.

54. Wiens K, Bhattarai A, Pedram P, Dores A, Williams J, Bulloch A, et al. A growing need for youth mental health services in Canada: examining trends in youth mental health from 2011 to 2018. Epidemiology and Psychiatric Sciences. 2020;29:e115.

55. Ren X, Yu S, Dong W, Yin P, Xu X, Zhou M. Burden of depression in China, 1990–2017: findings from the global burden of disease study 2017. J Affect Disord. 2020;268:95-101.

56. Thorisdottir IE, Asgeirsdottir BB, Sigurvinsdottir R, Allegrante JP, Sigfusdottir ID. The increase in symptoms of anxiety and depressed mood among Icelandic adolescents: time trend between 2006 and 2016. Eur J Public Health. 2017;27(5):856-61.

57. Sutcliffe K, Ball J, Clark TC, Archer D, Peiris-John R, Crengle S, et al. Rapid and unequal decline in adolescent mental health and well-being 2012–2019: Findings from New Zealand cross-sectional surveys. Aust N Z J Psychiatry. 2022;57(2):264-82.

58. Bobrowski K, Ostaszewski K, Pisarska A. Mental health of Warsaw middle school students. Mokotow Study 2004-2016. Psychiatr Pol. 2019;55 1:127-43.

59. Coley RL, O’Brien M, Spielvogel B. Secular trends in adolescent depressive symptoms: growing disparities between advantaged and disadvantaged schools. J Youth Adolesc. 2019;48(11):2087-98.

60. Duffy ME, Twenge JM, Joiner TE. Trends in mood and anxiety symptoms and suicide-related outcomes among U.S. undergraduates, 2007–2018: evidence from two national surveys. J Adolesc Health. 2019;65(5):590-8.

61. Goodwin RD, Weinberger AH, Kim JH, Wu M, Galea S. Trends in anxiety among adults in the United States, 2008-2018: rapid increases among young adults. J Psychiatr Res. 2020;130:441-6.

62. Keyes KM, Gary D, O’Malley PM, Hamilton A, Schulenberg J. Recent increases in depressive symptoms among US adolescents: trends from 1991 to 2018. Soc Psychiatry Psychiatr Epidemiol. 2019;54(8):987-96.

63. Lipson SK, Lattie EG, Eisenberg D. Increased rates of mental health service utilization by U.S. college students: 10-year population-level trends (2007-2017). Psychiatr Serv. 2019;70(1):60-3.

64. Lu W. Adolescent depression: national trends, risk factors, and healthcare disparities. Am J Health Behav. 2019;43(1):181-94.

65. Lu W, Kim JC, Yoon AS, Yun KK, Solomon P. Trends and disparities in unmet treatment needs for co-occurring depression and alcohol use disorders among young adults in the U.S. Am J Orthopsychiatry. 2022;92(3):268-79.

66. Parks MJ, Roesler J, Menanteau B, Raguet M, Eisenberg ME. The Intersection of Depressive Symptoms, Adverse Childhood Experiences, and Protective Factors Among Adolescents: Epidemiological Evidence from Minnesota, 2016 and 2019. Advers Resil Sci. 2022;3(1):21-36.

67. Parodi KB, Holt MK, Green JG, Porche MV, Koenig B, Xuan Z. Time trends and disparities in anxiety among adolescents, 2012–2018. Soc Psychiatry Psychiatr Epidemiol. 2022;57(1):127-37.

68. Twenge JM, Cooper AB, Joiner TE, Duffy ME, Binau SG. Age, Period, and Cohort Trends in Mood Disorder Indicators and Suicide-Related Outcomes in a Nationally Representative Dataset, 2005-2017. J Abnorm Psychol. 2019;128(3):185-99.

69. Wang C, Li K, Kim M, Lee S, Seo DC. Association between psychological distress and elevated use of electronic devices among U.S. adolescents: results from the youth risk behavior surveillance 2009-2017. Addict Behav. 2019;90:112-8.

70. Buli BG, Larm P, Nilsson KW, Hellström-Olsson C, Giannotta F. Trends in mental health problems among Swedish adolescents: Do school-related factors play a role? PLoS One. 2024;19(3):e0300294.

71. Ahn-Horst RY, Bourgeois FT. Mental Health-Related Outpatient Visits Among Adolescents and Young Adults, 2006-2019. JAMA Netw Open. 2024;7(3):e241468.

72. Baumgarten F, Junker S, Schlack R. Prevalence and Time Trends of Self-Reported Mental Health Problems Among Children and Adolescents Between 11 and 17 Years in the KiGGS Study. Z Kinder Jugendpsychiatr Psychother. 2023;51(4):311-20.

73. McGorry PD, Mei C, Amminger GP, Yuen HP, Kerr M, Spark J, et al. A Sequential Adaptive Intervention Strategy Targeting Remission and Functional Recovery in Young People at Ultrahigh Risk of Psychosis: The Staged Treatment in Early Psychosis (STEP) Sequential Multiple Assignment Randomized Trial. JAMA Psychiatry. 2023;80(9):875-85.

74. Burke T, Thompson A, Mifsud N, Yung AR, Nelson B, McGorry P, et al. Proportion and characteristics of young people in a first-episode psychosis clinic who first attended an at-risk mental state service or other specialist youth mental health service. Schizophr Res. 2022;241:94-101.

75. Australian Institute of Health and Welfare. Young Australians: their health and wellbeing 2007. Cat. no. PHE 87. Canberra; 2007.

76. Australian Institute of Health and Welfare. Young Australians: their health and wellbeing 2011. Cat. no. PHE 140. Canberra; 2011.

77. Australian Bureau of Statistics. National Study of Mental Health and Wellbeing. Available at: <https://www.abs.gov.au/statistics/health/mental-health/national-study-mental-health-and-wellbeing/2020-21> 2022 [

78. Jensen HAR, Davidsen M, Møller SR, Román JEI, Kragelund K, Christensen AI, et al. The health of the Danes: The national health profile 2021 [Danskernes sundhed – Den Nationale Sundhedsprofil 2021]. Copenhagen; 2022.

79. Thorisdottir IE, Asgeirsdottir BB, Kristjansson AL, Valdimarsdottir HB, Jonsdottir Tolgyes EM, Sigfusson J, et al. Depressive symptoms, mental wellbeing, and substance use among adolescents before and during the COVID-19 pandemic in Iceland: a longitudinal, population-based study. Lancet Psychiatry. 2021;8(8):663-72.

80. Cerniglia L, Cimino S. Eating Disorders and Internalizing/Externalizing Symptoms in Adolescents before and during the COVID-19 Pandemic. J Am Nutr Assoc. 2023;42(5):445-51.

81. van der Velden PG, van Bakel HJA, Das M. Mental health problems among Dutch adolescents of the general population before and 9 months after the COVID-19 outbreak: A longitudinal cohort study. Psychiatry Res. 2022;311:114528.

82. Burdzovic Andreas J, Brunborg GS. Self-reported mental and physical health among Norwegian adolescents before and during the COVID-19 pandemic. JAMA Network Open. 2021;4(8):e2121934.

83. Hafstad GS, Sætren SS, Wentzel-Larsen T, Augusti E-M. Adolescents' symptoms of anxiety and depression before and during the Covid-19 outbreak - a prospective population-based study of teenagers in Norway. Lancet Regional Health Europe. 2021;5:100093.

84. Gagné T, Schoon I, McMunn A, Sacker A. Mental distress among young adults in Great Britain: long-term trends and early changes during the COVID-19 pandemic. Soc Psychiatry Psychiatr Epidemiol. 2022;57(6):1261-72.

85. Niedzwiedz CL, Green MJ, Benzeval M, Campbell D, Craig P, Demou E, et al. Mental health and health behaviours before and during the initial phase of the COVID-19 lockdown: longitudinal analyses of the UK Household Longitudinal Study. J Epidemiol Community Health. 2021;75(3):224-31.

86. Pierce M, Abel KM, Muwonge J, Jr., Wicks S, Nevriana A, Hope H, et al. Prevalence of parental mental illness and association with socioeconomic adversity among children in Sweden between 2006 and 2016: a population-based cohort study. Lancet Public Health. 2020;5(11):e583-e91.

87. Hawes MT, Szenczy AK, Klein DN, Hajcak G, Nelson BD. Increases in depression and anxiety symptoms in adolescents and young adults during the COVID-19 pandemic. Psychol Med. in press.

88. Guo B-C, Chen Y-J, Huang W-Y, Lin M-J, Wu H-P. Psychological disorders and suicide attempts in youths during the pre-COVID and post-COVID era in a Taiwan pediatric emergency department. Front Psychol. 2023;14.

89. Mitiku KW, Tegegne E, Amsalu M, Habtegiorgis SD, Melaku B. Mental illness in children and its determinants in Ethiopia: A systematic review and meta-analysis, 2023. Clin Child Psychol Psychiatry. 2024;29(1):168-86.

90. Matsumoto N, Kadowaki T, Takanaga S, Shigeyasu Y, Okada A, Yorifuji T. Longitudinal impact of the COVID-19 pandemic on the development of mental disorders in preadolescents and adolescents. BMC Public Health. 2023;23(1):1308.

91. Prichett LM, Yolken RH, Severance EG, Carmichael D, Zeng Y, Lu Y, et al. COVID-19 and Youth Mental Health Disparities: Intersectional Trends in Depression, Anxiety and Suicide Risk-Related Diagnoses. Acad Pediatr. 2024;24(5):837-47.

92. Reyes MP, Song I, Bhatt A. Breaking the Silence: An Epidemiological Report on Asian American and Pacific Islander Youth Mental Health and Suicide (1999–2021). Child and Adolescent Mental Health. 2024;29(2):136-44.

93. Wong SMY, Chen EYH, Suen YN, Wong CSM, Chang WC, Chan SKW, et al. Prevalence, time trends, and correlates of major depressive episode and other psychiatric conditions among young people amid major social unrest and COVID-19 in Hong Kong: a representative epidemiological study from 2019 to 2022. Lancet Reg Health West Pac. 2023;40:100881.
